# Safety, pharmacokinetics, and biological activity of CD4-mimetic BNM-III-170 in SHIV-infected rhesus macaques

**DOI:** 10.1128/jvi.00062-25

**Published:** 2025-04-07

**Authors:** Elise G. Viox, Jonathan Richard, Andres G. Grandea, Kevin Nguyen, Justin Harper, James Auger, Shilei Ding, Romain Gasser, Jérémie Prévost, Lorie Marchitto, Halima Medjahed, Catherine Bourassa, Fleur Gaudette, Amélie Pagliuzza, Cesar Ariel Trifone, Christina Gavegnano, Selwyn J. Hurwitz, Jun Park, Natasha M. Clark, Iman Hammad, Saverio Capuano, Malcolm A. Martin, Raymond F. Schinazi, Guido Silvestri, Deanna A. Kulpa, Priti Kumar, Nicolas Chomont, Marzena Pazgier, Amos B. Smith, Joseph Sodroski, David T. Evans, Andrés Finzi, Mirko Paiardini

**Affiliations:** 1 Division of Microbiology and Immunology, Emory National Primate Research Center, Emory University, Atlanta, Georgia, USA; 2 Centre de Recherche du CHUM, Montréal, Québec, Canada; 3 Département de Microbiologie, Infectiologie et Immunologie, Université de Montréal, Montréal, Québec, Canada; 4 Department of Pathology and Laboratory Medicine, University of Wisconsin-Madison 5228, Madison, Wisconsin, USA; 5 Department of Pediatrics, Laboratory of Biochemical Pharmacology, Emory Center for AIDS Research, Emory University School of Medicine and Children’s Healthcare of Atlanta, Atlanta, Georgia, USA; 6 Department of Chemistry, School of Arts and Sciences, University of Pennsylvania 6572, Philadelphia, Pennsylvania, USA; 7 Laboratory of Molecular Microbiology, National Institute of Allergy and Infectious Diseases, National Institutes of Health 35037, Bethesda, Maryland, USA; 8 Department of Pathology and Laboratory Medicine, School of Medicine, Emory University 12239, Atlanta, Georgia, USA; 9 Department of Internal Medicine, Section of Infectious Diseases, Yale University School of Medicine 198963 , New Haven, Connecticut, USA; 10 Infectious Diseases Division, Department of Medicine, Uniformed Services University of the Health Sciences 1685, Bethesda, Maryland, USA; 11 Department of Microbiology, Harvard Medical School, Boston, Massachusetts, USA; 12 Department of Cancer Immunology and Virology, Dana-Farber Cancer Institute 1855, Boston, Massachusetts, USA; 13 Wisconsin National Primate Research Center, University of Wisconsin-Madison 5228, Madison, Wisconsin, USA; St. Jude Children's Research Hospital, Memphis, Tennessee, USA

**Keywords:** human immunodeficiency virus, nonhuman primate, ADCC, CD4 mimetic, in vivo therapeutic strategies

## Abstract

Anti-HIV-1 antibodies capable of mediating ADCC are elicited by the majority of people with HIV-1 and preferentially target the “open,” CD4-bound conformation of HIV-1 envelope glycoproteins (Env). However, due to the “closed” conformation sampled by unliganded HIV-1-Envs, these antibodies are ineffective at eliminating infected cells. BNM-III-170 is a small-molecule CD4-mimetic compound that binds the Phe43 cavity of the gp120 subunit of Env, forcing Env to “open up,” thus exposing epitopes targeted by CD4-induced (CD4i), ADCC-mediating antibodies. Here, we assessed the safety, pharmacokinetics, and biological activity of BNM-III-170 in uninfected and SHIV-AD8-EO-infected rhesus macaques (RMs). In uninfected RMs, single subcutaneous administrations of 3–36 mg/kg BNM-III-170 were well-tolerated, with serum half-lives ranging from 3 to 6 h. In SHIV-infected RMs, four different regimens were evaluated: 2 × 36 mg/kg daily, 1 × 24 mg/kg, 3 × 36 mg/kg every 7 days, and 3 × 36 mg/kg every 3 days. While toxicity was observed with daily doses, all other regimens demonstrated reasonable safety profiles. No changes in plasma viral loads were observed in SHIV-infected RMs following any of the evaluated BNM-III-170 dosing regimens. However, plasma collected following BNM-III-170 administration was shown to have increased binding to infected cells and to sensitize SHIV AD8-EO virions to neutralization by otherwise non-neutralizing antibodies. In addition, the plasma of treated animals mediated ADCC in the presence of BNM-III-170. These results establish a well-tolerated BNM-III-170 dosing regimen in SHIV-infected RMs and serve as proof of concept for its biological activity in promoting the targeting of infected cells by CD4i ADCC-mediating antibodies. Thus, they inform future studies evaluating CD4mc treatment in ART-treated animals.

**IMPORTANCE:**

A therapeutic regimen able to eradicate or functionally cure HIV-1 remains elusive and may require a “shock-and-kill” approach to reactivate and then purge the latent HIV-1 reservoir. The small-molecule CD4-mimetic compound BNM-III-170 has previously been shown to (i) sensitize HIV-1-infected cells to ADCC mediated by plasma from people with HIV-1 (PWH) *in vitro* and (ii) significantly delay the time to viral rebound following ART interruption when combined with anti-CoRBS + anti-cluster A Abs or plasma from PWH in humanized mice. To evaluate the use of BNM-III-170 as part of a kill approach, we characterized the safety, pharmacokinetics, and biological activity of BNM-III-170 in uninfected and SHIV-infected RMs. Our study identifies a tolerable BNM-III-170 dosing regimen in SHIV-infected RMs and provides insights into its antiviral activities; as such, it informs future studies evaluating the efficacy of BNM-III-170 in reducing the viral reservoir.

## INTRODUCTION

There are approximately 40 million PWH, with 1.3 million new infections occurring in 2023 (1). While combination antiretroviral therapy (ART) effectively controls viral replication, cessation of ART results in viral rebound, due to the persistence of replication-competent HIV-1 in a reservoir of infected CD4+ T cells (2–9). The development of a therapeutic regimen capable of functionally curing HIV-1 infection would have a profound impact on public health. Recent efforts to achieve a cure for HIV-1 have focused on “shock-and-kill” approaches aimed at inducing HIV-1 transcription and protein expression in latently infected cells with latency-reversing agents (LRAs) and killing reactivated cells through immune-mediated clearance (10). Several LRAs, including histone deacetylase (HDAC) inhibitors vorinostat and romidepsin, have been shown to induce viral production in PWH on ART. However, these infected cells were not efficiently cleared following reactivation (11–13). To promote the killing of latently infected cells in which viral transcription and protein production have been induced, it is likely that immune-based strategies must be pursued.

A promising strategy for eliminating HIV-1-infected cells following viral reactivation is through antibody-dependent cellular cytotoxicity (ADCC). ADCC is an immune mechanism in which Fc-receptor-bearing monocytes and NK cells recognize and actively lyse antibody-coated target cells. HIV-1-specific antibodies (Abs) with ADCC effector functions are commonly elicited in natural HIV-1 infection and have been detected as early as 1 month following infection (14–16). The primary target of HIV-1-specific Abs is the envelope glycoprotein (Env) trimer, which is expressed on the surface of HIV-1-infected cells and HIV-1 virions. HIV-1-specific Abs naturally elicited in PWH are capable of mediating potent ADCC, provided Env is present in its “open” CD4-bound conformation (17). Among these CD4-induced (CD4i) Abs, gp120-specific, anti-cluster A Abs exhibit the highest activity (18–21). However, due to (i) the “closed” conformation of unliganded Envs from primary HIV-1 strains and (ii) HIV-1 Vpu and Nef-mediated downmodulation of surface CD4, which precludes Env-CD4 interaction (22), these Abs are ineffective in ADCC-mediated clearance of infected cells (17, 23). Small-molecule CD4-mimetic compounds (CD4mc), such as BNM-III-170, target the conserved gp120 Phe43 cavity (24, 25) and were shown to “open up” Env. This sensitizes HIV-1-infected cells to ADCC mediated by plasma from PWH (16) and CD4i Abs including anti-cluster A and anti-coreceptor binding site (CoRBS) Abs (26).

The therapeutic efficacy of BNM-III-170 has previously been evaluated *in vivo* in HIV-1-infected SRG-15 humanized mice with promising results (27). In viremic, HIV-1-infected SRG-15 Hu-PBL mice, co-administration of BNM-III-170 with a combination of anti-CoRBS and anti-cluster A Abs or plasma from PWH was shown to reduce HIV-1 DNA levels in CD4+ T cells isolated from peripheral blood, spleen, mucosa, and liver. In addition, HIV-1-infected, ART-treated SRG-15 Hu-HSC mice that received BNM-III-170 with a combination of anti-CoRBS and anti-cluster A Abs or plasma from PWH at analytic treatment interruption (ATI) experienced a significant delay in time to viral rebound as compared to mock-treated mice.

Non-human primates are physiologically and genetically similar to humans and represent important animal models for HIV-1 pathogenesis, vaccine, and cure research. While Madani et al*.* previously challenged HIV-1 gp120-immunized RMs with SHIV mixed with BNM-III-170, no prior studies evaluating the therapeutic efficacy of BNM-IIII-170 in chronically infected RMs have been conducted (28).

Here, we first conducted dose-escalation studies of BNM-III-170 in uninfected RMs and then evaluated the safety, pharmacokinetics, and biological activity of BNM-III-170 in four chronically SHIV AD8-EO-infected RMs. While this study was limited in size, we successfully established a well-tolerated BNM-III-170 dosing regimen in SHIV AD8-EO-infected RMs and demonstrated the biological activity of bioavailable BNM-III-170, informing future studies evaluating the ability of CD4mc to reduce the viral reservoir.

## RESULTS

### Single subcutaneous doses of 3–36 mg/kg BNM-III-170 were safe and well tolerated in uninfected RMs

Single-dose administrations of 3–36 mg/kg BNM-III-170 were first evaluated in uninfected rhesus macaques (Table S1). BNM-III-170 was synthesized using an improved enantioselective route (Fig. S1) and administered at a concentration of 13.5 mg/mL in a 20% DMSO solution. Initially, one intravenous 3 mg/kg dose of BNM-III-170 was evaluated in a single RM and resulted in an anaphylactic-like reaction characterized by vomiting, tachycardia, and hypotension that resolved following epinephrine treatment. In addition, at 24 h post-BNM-III-170 administration, elevated creatine phosphokinase (CPK) (5,364 U/L, normal range: 115–544 U/L), aspartate transferase (AST) (334 U/L, normal range: 23–85 U/L), and alanine transaminase (ALT) (221 U/L, normal range: 18–90 U/L) were observed in this animal; these enzymes returned to baseline levels within 1 week of treatment (Table 1). Since intravenous administration of drugs is prone to elicit anaphylactic-like reactions, administration of BNM-III-170 via a subcutaneous route was next explored (29). While the 3 mg/kg subcutaneous (SQ) dose of BNM-III-170 administered to a second RM was well tolerated with no anaphylactic-like reactions, transient elevations of CPK (5053 U/L) and AST (106 U/L) were similarly observed following BNM-III-170 administration.

**TABLE 1:** Safety profile of BNM-III-170 in uninfected rhesus macaques^*a*^

BNM-III-170 dose (mg/kg)	Route of administration	No. of RMs	Animal ID	Clinical signs	Blood chemistries
3	Intravenous	1	r10019	Vomiting, tachycardia, hypotension	Transient elevation of CPK, AST, ALT
3	Subcutaneous	1	r12047	Normal	Transient elevation of CPK, AST
6	Subcutaneous	1	r12047	Normal	Normal
12	Subcutaneous	1	r10019	Normal	Transient elevation of CPK, AST, ALT
14	Subcutaneous	4	r10019	Normal	Transient elevations of CPK, AST, ALT
			r12047	Normal	Normal
			r14094	Normal	Transient elevation of AST
			r14131	Normal	Transient elevations of AST, ALT
24	Subcutaneous	2	r10019	Normal	Transient elevations of CPK, AST, ALT, troponin I
			r12047	Normal	Normal
36	Subcutaneous	2	r14094	Normal	Transient elevations of CPK, AST
			r15023	Normal	Transient elevation of CPK, AST, ALT, troponin I^*b*^

^*a*^ Subcutaneous BNM-III-170 administration at doses up to 36 mg/kg is safe and well tolerated in uninfected RMs.

^*b*^ In this animal, a transient increase in the serum level of troponin I was also seen after the administration of intramuscular ketamine without the administration of subcutaneous BNM-III-170.

Of note, prior to receiving BNM-III-170, all RMs were anesthetized with an intramuscular injection of ketamine. Since muscle injury due to injection has been shown to increase CPK, AST, and ALT, these elevated enzymes may have been due to ketamine administration (30, 31). However, elevated AST and ALT levels can also indicate hepatotoxicity, and elevated CPK and AST can result from cardiac muscle damage (32–34). Previously, a different CD4mc referred to as 2 HCl was shown to be cardiotoxic in a RM, resulting in prolonged PR interval and bradycardia (35). To rule out the possibility that the elevated CPK and AST in our uninfected RMs were due to BNM-III-170 cardiotoxicity, we added a more specific marker for cardiac damage, troponin I, to the serum chemistry panel for all subsequent doses.

In subsequent dose-escalation studies, RMs received single SQ BNM-III-170 doses of 6 mg/kg (*n* = 1), 12 mg/kg (*n* = 1), 14 mg/kg (*n* = 4), 24 mg/kg (*n* = 2), and 36 mg/kg (*n* = 2). Of note, when multiple doses of BNM-III-170 were evaluated in the same animal, each treatment was spaced out by at least 2 months to ensure BNM-III-170 washout. Notably, 6–36 mg/kg SQ doses of BNM-III-170 were well tolerated, and no clinical signs of toxicity were observed. Similar to what was observed in the animals treated with 3 mg/kg BNM-III-170, transient increases in CPK, AST, and ALT were seen in most animals treated with 6–36 mg/kg BNM-III-170. In one of the RMs receiving 24 mg/kg BNM-III-170 and one of the RMs receiving 36 mg/kg, low-level transient increases in serum troponin I (0.068 and 0.055 ng/mL, respectively, normal range: ≤0.041) were observed at 24 h post-BNM-III-170 administration; these troponin I levels returned to baseline levels by 4 days post-treatment. Of note, the RM that had elevated troponin I following a 36 mg/kg dose of BNM-III-170 also experienced transient low-level increases in troponin I following intramuscular injection of ketamine without BNM-III-170 (0.021 ng/mL). The diagnosis of acute myocardial infarction requires elevated troponin I levels sustained for several days and electrocardiogram changes indicative of ischemia (new ST-T wave changes, new left bundle branch block, or the development of new pathologic Q waves) (36). No electrocardiogram abnormalities were observed in any of the monkeys receiving subcutaneous BNM-III-170. Therefore, the transient nature of the observed troponin I elevations and the absence of electrocardiogram changes make it highly unlikely that either of the monkeys receiving the higher doses of BNM-III-170 experienced an acute myocardial infarction. In summary, while transient elevations in CPK, AST, and ALT were observed following subcutaneous administration of 3–36 mg/kg BNM-II-170, subcutaneous doses of 3–36 mg/kg BNM-III-170 were shown to be tolerable in uninfected RMs.

### Bioavailable BNM-III-170 in treated, uninfected RMs increases anti-CoRBS Ab binding to infected cells

**Fig 1:**
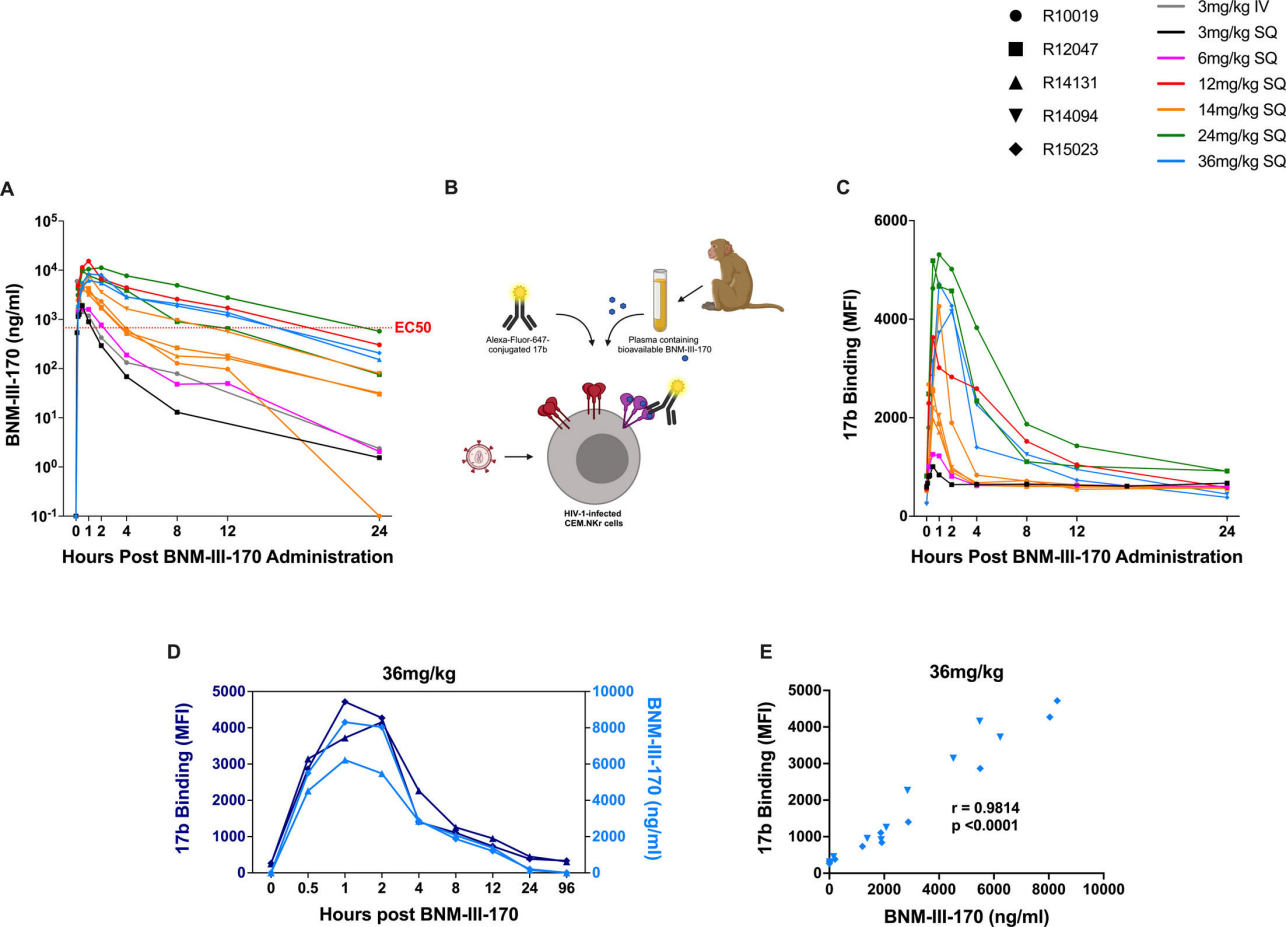
BNM-III-170 pharmacokinetics in uninfected RMs and functional activity of bioavailable BNM-III-170. (**A**) Plasma levels of BNM-III-170 following intravenous administration of 3 mg/kg BNM-III-170 and subcutaneous administration of 3–36 mg/kg BNM-III-170 in uninfected RMs. (**B**) Experimental setup of the assay to measure binding of anti-CoRBS mAb 17b to Env expressed on CH058TF-infected cells following incubation with RM plasma containing bioavailable BNM-III-170. (**C**) Binding of 17b to CH058TF-infected cells following incubation with plasma collected from uninfected RMs at selected timepoints after subcutaneous 3–36 mg/kg BNM-III-170 administration. (**D**) 17b binding and bioavailable BNM-III-170 in plasma from select timepoints following subcutaneous administration of 36 mg/kg BNM-III-170. (**E**) Association between plasma levels of BNM-III-170 in RMs that received one SQ 36 mg/kg dose of BNM-III-170 and 17b binding to CH058TF-infected cells following incubation with plasma collected after SQ 36 mg/kg BNM-III-170 administration (Spearman correlation, *n* = 22).

To evaluate the pharmacokinetics of BNM-III-170 in uninfected RMs, a methodology for the detection of as little as 1.95 nM BNM-III-170 in plasma was established by liquid chromatography and mass spectrometry. Plasma levels of BNM-III-170 were observed to peak between 30 minutes to 2 h post-administration in treated animals, reaching max concentrations of 1.9–15.3 µg/mL (Fig. 1A). The highest maximum plasma concentrations of BNM-III-170 were observed with 12 mg/kg BNM-III-170 (C_max_: 15.3 µg/mL) and 24 mg/kg BNM-III-170 (C_max_: 11.2 µg/mL and 9.7 µg/mL). One potential explanation for the 12 mg/kg and 24 mg/kg BNM-III-170 doses resulting in higher maximum plasma BNM-III-170 concentrations than the 36 mg/kg dose is animal variability in drug metabolism; while the 36 mg/kg dose was evaluated in animals R14094 and R15023, the 12 mg/kg dose was tested in animal R10019 and the 24 mg/kg dose in animals R10019 and R12047. Pharmacokinetic analyses from these dose-escalation studies indicated a BNM-III-170 plasma half-life of 3–6 h following SQ administration. Of note, BNM-III-170 levels above the estimated effective concentration for ADCC of 0.675 µg/mL (37) were maintained for 8–12 h at doses of 12 mg/kg, 24 mg/kg, and 36 mg/kg, indicating that repeated dosing may be necessary for sustaining therapeutic levels of BNM-III-170 in RMs.

Next, we assessed the ability of the plasma collected from uninfected RMs at various timepoints following BNM-III-170 administration to induce conformational changes in Env expressed on the surface of HIV-1-infected cells. Briefly, plasma was incubated with cells infected with the transmitted/founder virus CH058 (CH058TF) and the binding of the Alexa-Fluor-647-conjugated anti-CoRBS mAb 17b (whose epitope is not exposed on the “closed” Env conformation) to Env expressed on infected cells was measured by FACS (Fig. 1B). BNM-III-170 treatment was consistently shown to increase 17b binding across all tested doses, peaking at 30 minutes to 2 h post-administration (Fig. 1C). The kinetic curves for 17b binding closely paralleled the plasma levels of BNM-III-170, with plasma from each dose and all combined doses showing a positive correlation between 17b binding and BNM-III-170 plasma concentrations (Fig. 1D and E; Fig. S2A through F). Importantly, residual 17b activity was seen up to 8 h after the subcutaneous administration of BNM-III-170 at the highest tested dose (Fig. 1D); these results suggest that, even when plasma BNM-III-170 levels are near the limit of detection, its biological activity might remain.

### Safety profile of BNM-III-170 in SHIV-infected RMs

**Fig 2:**
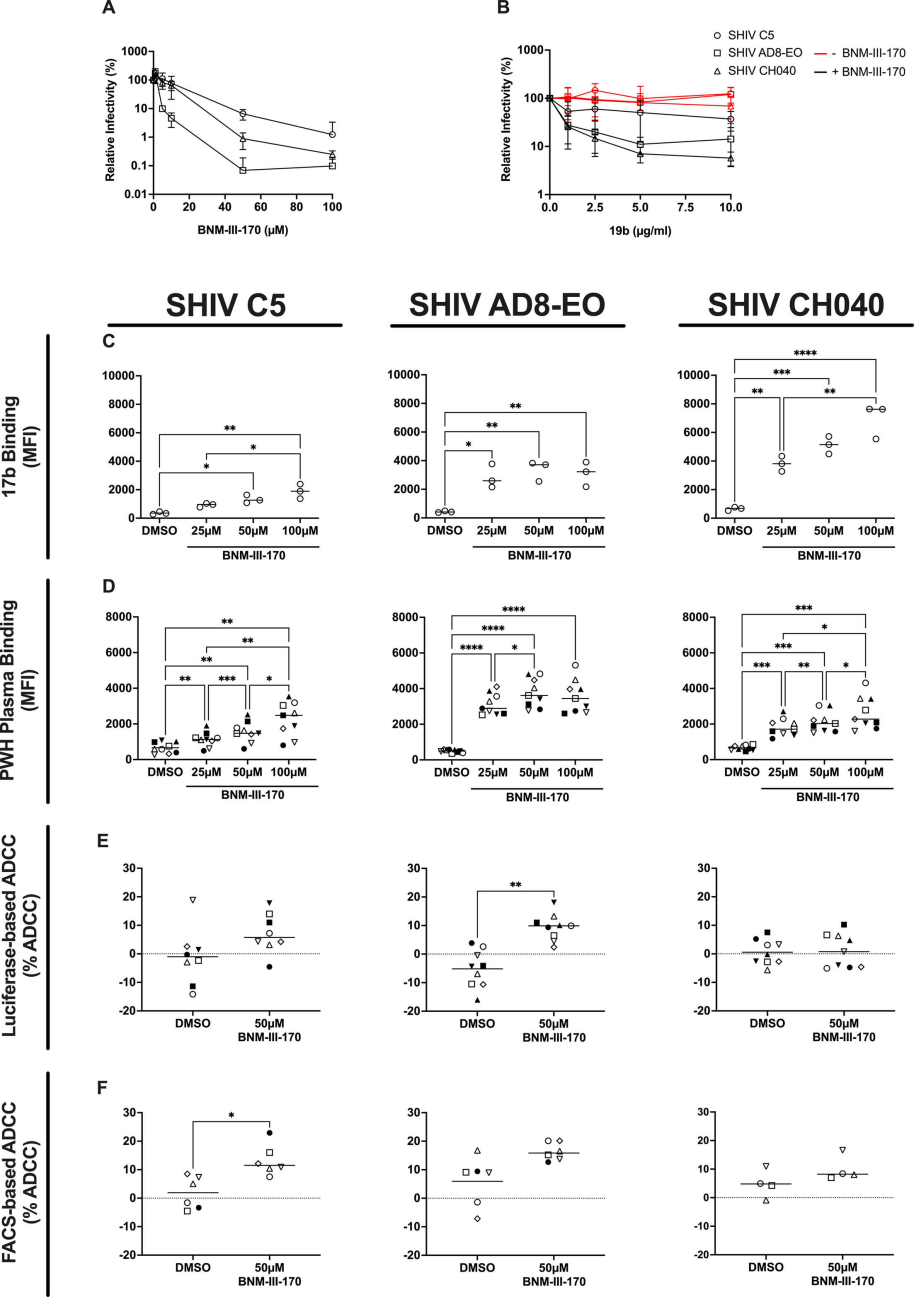
Biological activity of BNM-III-170 against SHIV variants. (**A**) Infectivity of SHIV variants when incubated with the indicated concentrations of BNM-III-170. (**B**) Infectivity of SHIV variants when incubated with non-neutralizing CD4i antibody 19b in the presence or absence of 2 µM BNM-III-170. (**C**) 17b binding to CEM.NKr-CCR5-sLTR-Luc cells infected with SHIV variants in the absence (DMSO) or presence of the indicated concentrations of BNM-III-170 from three independent experiments. Statistical analyses were performed using Kruskal-Wallis with Dunnett’s multiple comparisons tests. (**D**) Binding of plasma from PWH (*n* = 9) to CEM.NKr-CCR5-sLTR-Luc cells infected with SHIV variants in the absence (DMSO) or presence of the indicated concentrations of BNM-III-170. Statistical analyses were performed using repeated-measures one-way ANOVA with Tukey’s multiple comparisons tests. Percent ADCC-mediated killing of CEM.NKr or primary CD4 T cells infected with SHIV variants in the absence (DMSO) or presence of 50 µM BNM-III-170 as measured by (**E**) luciferase-based and (**F**) FACS-based ADCC assays, respectively. Statistical analyses were performed using ordinary ANOVA with Dunnett’s multiple comparisons tests in C-D and Wilcoxon matched-pairs signed rank test in E-F. *P-value < 0.05, **P-value < 0.01, ***P-value < 0.001, ****P-value < 0.0001.

Following the completion of the safety and pharmacokinetic study in uninfected RMs, we proceeded with a pilot study in SHIV-infected RMs to establish a well-tolerated BNM-III-170 dosing regimen in viremic animals. Three SHIVs were evaluated for potential use in this pilot study: SHIV C5, SHIV AD8-EO, and SHIV CH40. SHIV AD8-EO was ultimately selected for *in vivo* studies based on its ability to establish persistent infections in RMs and the combined abilities of BNM-III-170 to (i) directly decrease SHIV AD8-EO infectivity (Fig. 2A), (ii) sensitize SHIV AD8-EO to neutralization by the otherwise non-neutralizing, anti-V3 CD4i 19b antibody (Fig. 2B), (iii) induce conformational changes that exposed the 17b mAb epitope (Fig. 2C) and enhance binding of plasma from PWH (Fig. 2D), and (iv) sensitize target cells infected with SHIV AD8-EO to ADCC in the presence of plasma from PWH, as measured by luciferase- and FACS-based ADCC assays (Fig. 2E and F).

**Fig 3:**
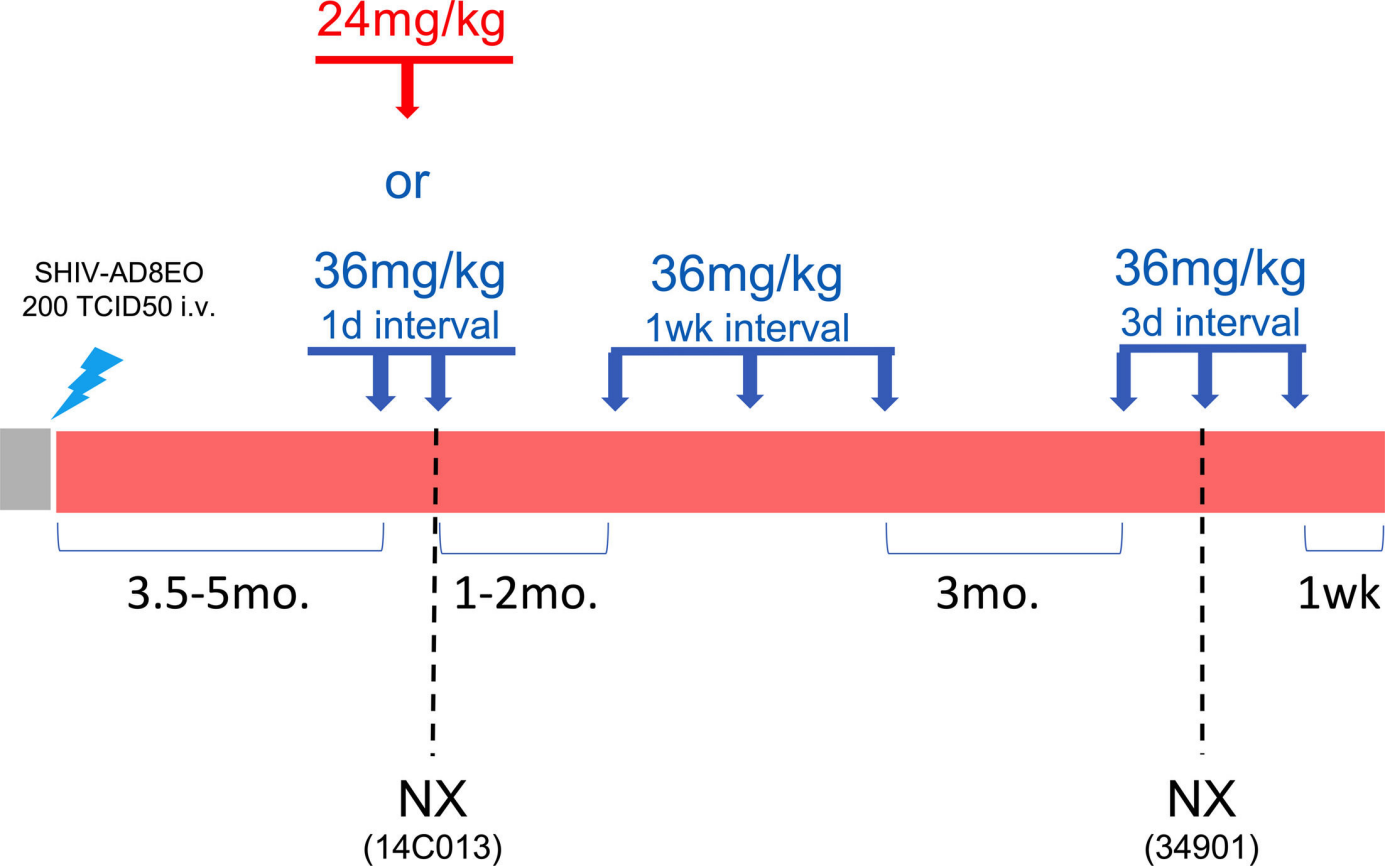
Summary of study design of subcutaneous BNM-III-170 treatment in SHIV AD8-EO-infected RMs. 36 mg/kg and 24 mg/kg doses of BNM-III-170 are represented by blue and red arrows, respectively. Two animals underwent necropsy (NX) due to an adverse event of unknown cause (animal 14C013) or weight loss due to diarrhea that started prior to BNM-III-170 dosing (animal 34901).

Four adult female RMs (4–5 years old, average 4.5 years) were infected intravenously with 200 TCID_50_ of SHIV AD8-EO and divided into two collection groups (Gr. 1: animals 14C013 and RYj17, Gr. 2: animals 14C056 and 34901) (Fig. 3; Fig. S3). All four animals exhibited peak plasma viral loads between 10^7^ and 10^8^ copies/mL and established set point viral loads between 10^4^ and 10^6^ copies/mL plasma (Fig. S4). RMs received their first treatment cycle of BNM-III-170 following the establishment of a stable set point viral load, at approximately 3.5–5 months post-infection. First, the two animals in Gr. 1 (14C013 and RYj17) received 2 × 36 mg/kg BNM-III-170 SQ at a dosing interval of once every 24 h. At the 6 h collection timepoint following the second dose, animal 14C013 was noted to have pale mucus membranes, weak femoral pulses, and dyspnea (Table 2). Physical examination with bloodwork and X-rays were performed. Excessive gas in the gastrointestinal tract was observed, but no other significant findings were noted that explained the pallor, cardiovascular abnormalities, or dyspnea. After examination, the animal failed to fully recover from sedation and was euthanized. At necropsy, animal 14C013 exhibited elevated troponin I (0.28 ng/mL), AST (145 U/L), ALT (123 U/L), amylase (421 U/L; normal range: 148–316 U/L), and a pale liver, suggesting liver injury, but the cause of the adverse reaction could not be established. While no clinical symptoms were observed in the other treated animal, RYj17, X-rays performed 24 h after the second dose showed an enlarged liver. In addition, RYj17 was found to have slightly elevated troponin I at 6 h after dose 1 (0.049 ng/mL) that resolved by 24 h after dose 1 and elevated ALP at 24 h after dose 2 that remained elevated 16 days later (405 and 715 U/L, respectively; normal range: 83–363 U/L).

**TABLE 2:** Safety profile of subcutaneous BNM-III-170 in SHIV AD8-EO-infected RMs

BNM-III-170 dose (mg/kg)	No. of BNM-III-170 doses	BNM-III-170 dosing interval	No. of RMs	Animal ID	Clinical signs	Blood chemistries
36	2	1 day	2	14C013	Animal experienced acute respiratory distress and tachycardia at 6 hr post dose #2 and was sacrificed; inconclusive path report, pale liver noted	Elevated AST, ALT, amylase, CPK, troponin I
				RYj17	Enlarged liver and spleen, lesion at BNM-III-170 injection site	Transient elevation of ALP, CPK, troponin I
24	1	NA	2	14C056		Transient elevation of CPK
				34901		Transient elevation of CPK, troponin I
36	3	7 days	3	RYj17		Transient elevation of AST, ALP, TRIG, CPK
				14C056		Transient elevation of AST, ALT, ALP, CSK
				34901		Transient elevation of Glob, ALT, ALP, GGT, AMY, CPK
36	3	3 days	2	RYj17	Mild erythema at injection sites	Transient elevation of ALT, CPK
				14C056	Mild erythema at injection sites	Transient elevation of AST, ALT, CPK
				34901	Animal reached endpoint criteria (weight loss > 25%) due to chronic diarrhea that started prior to CD4mc treatments, animal sacrificed 3 hr post dose #2	Elevated BUN, BUN/Cr, CPK; low potassium

To determine if a single, lower dose of BNM-III-170 may be more tolerated in SHIV-infected RMs, animals in Group 2 (14C056 and 34901) received 1 × 24 mg/kg SQ dose of BNM-III-170 for their first treatment cycle. No adverse reactions were observed in these animals. Animal 34901 exhibited elevated troponin I at 6 h post-dose (0.090 ng/mL) that resolved by the next tested timepoint at 5 days post-treatment. Next, we evaluated if 36 mg/kg BNM-III-170 doses were better tolerated when administered at a dosing interval greater than 24 h. One to two months following their respective initial BNM-III-170 treatment cycles, the three remaining animals (RYj17, 14C056, and 34901) received 36 mg/kg doses at a 7-day dosing interval. Transient increases in liver enzymes were observed following BNM-III-170 treatment, but no clinical signs of toxicity were noted. Given the short half-life of BNM-III-170, we evaluated the tolerability of 36 mg/kg BNM-III-170 doses administered at a shorter dosing interval of once every 3 days. Animals RYj17 and 14C056 received three doses during this treatment cycle. Both animals displayed mild erythema at the BNM-III-170 injection sites 24 h after dose 2. In addition, at 24 h after dose 3, RYj17 and 14C056 had elevated ALT (131 and 125), and 14C056 also displayed elevated AST (109 U/L) that resolved by the next anesthetic access 5 days later. Due to weight loss resulting from chronic, treatment-resistant diarrhea that started prior to BNM-III-170 dosing, animal 34901 was sacrificed 3 h following the second BNM-III-170 dose. At 0 h of the treatment cycle and necropsy, 34901 exhibited elevated blood urea nitrogen (34 and 28 mg/Dl, respectively; normal range: 9–27 mg/DL), a high ratio of blood urea nitrogen to creatinine (37.8 and 35, respectively; normal range: 12.5–33.3), and low potassium (2.8 and 2.4 mmol/L, respectively; normal range: 3–5 mmol/L); these changes in blood chemistries are often associated with dehydration and were likely due to prolonged diarrhea (38).

**Fig 4:**
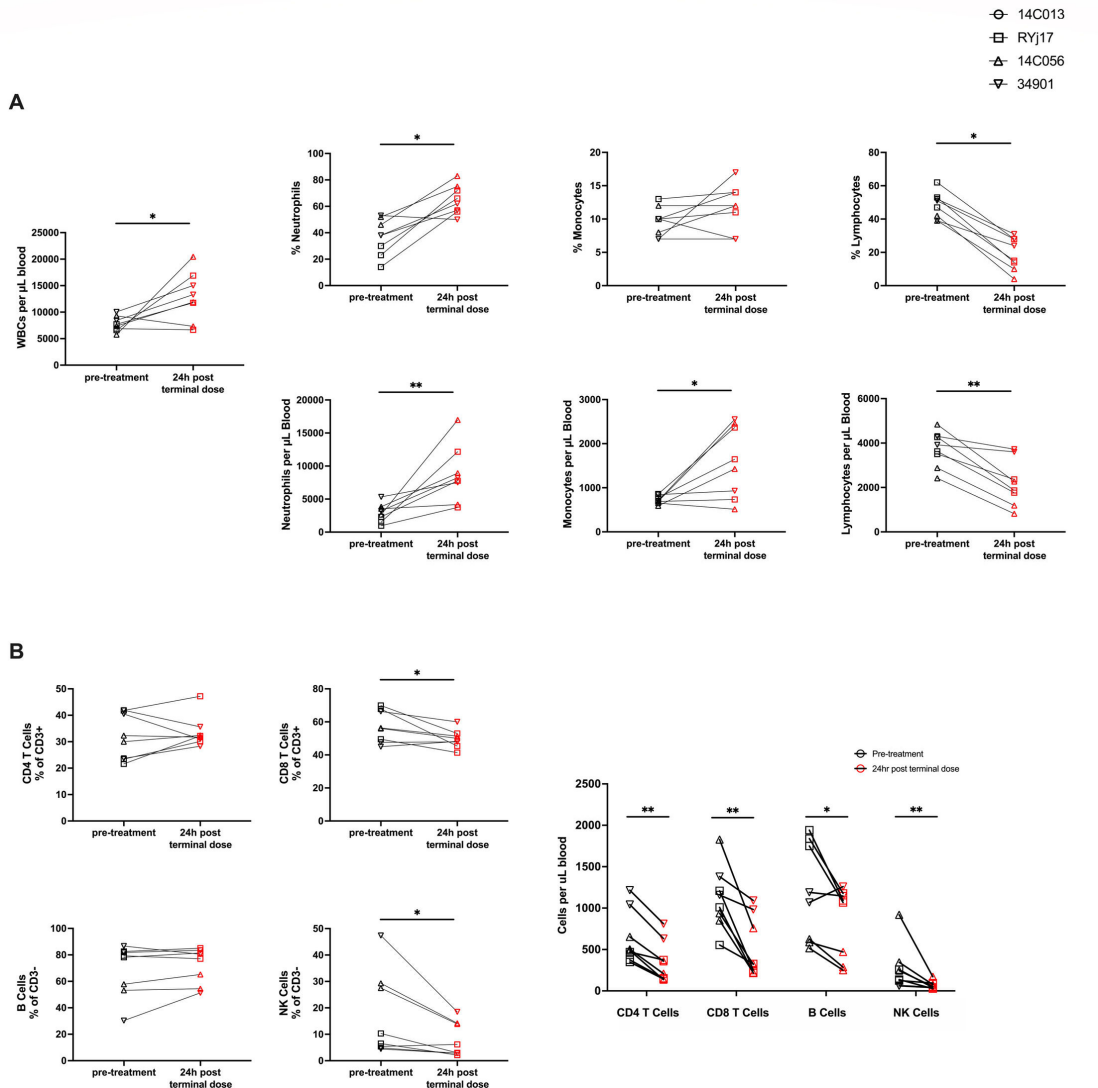
BNM-III-170 administration in viremic, SHIV AD8-EO-infected RMs results in increased neutrophils and monocytes and a non-specific reduction in lymphocytes. (**A**) WBC counts and neutrophil, lymphocyte, and monocyte percentages and counts in peripheral blood as determined by CBC panels. (**B**) Frequencies and absolute counts of CD4 T cells, CD8 T cells, B cells, and NK cells in PBMCs as determined by multi-color flow cytometry. CD4 T-cell and CD8 T-cell frequencies were reported as percentages of CD3+ lymphocytes and B-cell and NK cell frequencies as percentages of CD3- lymphocytes. Absolute counts were reported as cells per μL blood. Statistical analyses were performed using Wilcoxon matched-pairs signed rank tests comparing counts/frequencies from 0 h of the first dose and 24 h post-terminal dose for each treatment cycle for all animals. *P-value < 0.05, **P-value < 0.01.

In addition to monitoring blood chemistries, complete blood counts (CBCs) were performed immediately prior to BNM-III-170 treatment and at several post-treatment timepoints (Fig. 4A). Significant increases in white blood cell counts (WBCs) were observed between 0 h and 24 h after the terminal dose of each treatment cycle (median WBCs: 7,550 and 12,595, respectively, *P*-value = 0.0391). This increase in WBCs was largely attributed to increases in neutrophils (median counts at 0 h vs. 24 h of 2,926 and 8,004, *P*-value = 0.0078) and, to a lesser extent, monocytes (median counts at 0 h vs 24 h after the terminal dose of 696 and 1,537, *P*-value = 0.0391). Interestingly, there was also a significant decrease in lymphocyte frequency (49% at 0 h vs. 19.5% at 24 h post-terminal dose, *P* = 0.0078) and lymphocyte numbers (3,774 at 0 h vs. 2,068 at 24 h post-terminal dose, *P* = 0.0078) in whole blood. Of note, this lymphopenia was generally transient (Fig. S5).

To further investigate this observed lymphopenia, multiparameter flow cytometry was performed on PBMCs from 0 h and 24 h post-terminal dose timepoints from all treatment cycles (Fig. S6). Of note, the observed reduction in lymphocytes was found to be non-specific, with decreases in the absolute numbers of CD4+ T cells (median 496 vs. 283, *P* = 0.0078), CD8+ T cells (median 1,084 vs. 323.5, *P* = 0.0078), B cells (median 1,129 vs. 1,080, *P* = 0.0391), and NK cells (median 191 vs. 48, *P* = 0.0078) (Fig. 4B). While the exact cause of this non-specific lymphopenia is unknown, it may be due to depletion of lymphocytes or migration of lymphocytes to tissue; the latter explanation is more consistent with the transient nature of the lymphopenia.

In summary, while an adverse event of unknown cause was observed with daily BNM-III-170 dosing, all other tested BNM-III-170 dosing regimens were well-tolerated. Elevated liver enzymes, increases in WBCs, neutrophils, and monocytes, and non-specific lymphopenia were frequently observed following BNM-III-170 administration. While these changes were generally transient in the animals in this study, future studies evaluating BNM-III-170 should carefully monitor liver function and CBCs.

### Pharmacokinetics and biological activity of BNM-III-170 in SHIV-infected RMs

**Fig 5:**
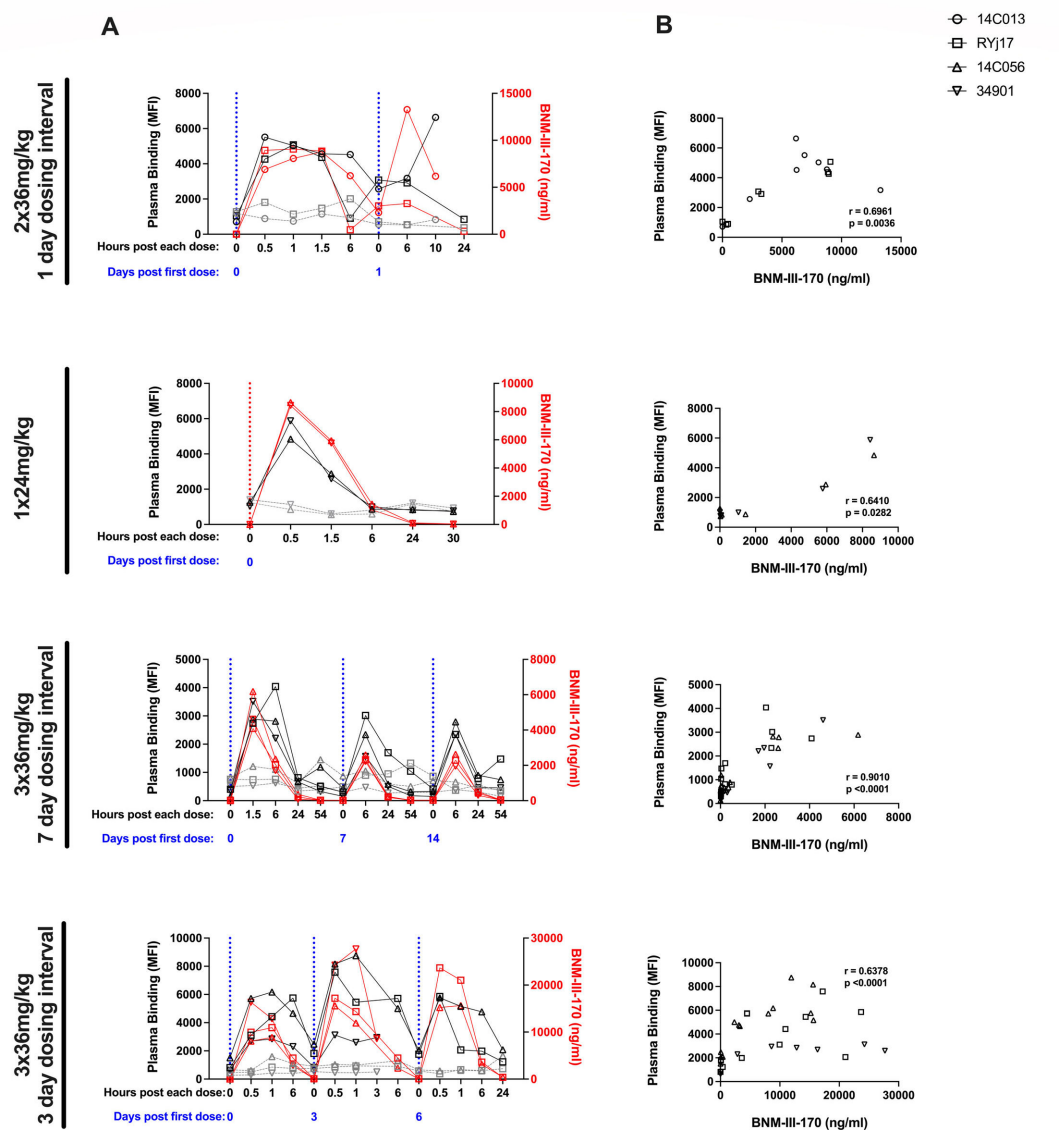
Bioavailable BNM-III-170 in plasma of treated, SHIV AD8-EO-infected RMs is correlated with increased plasma binding to HIV-1-infected cells. (**A**) Plasma binding and bioavailable BNM-III-170 in plasma during each BNM-III-170 treatment cycle. Plasma binding to mock-infected (gray) or CH058TF-infected (black) CEM.NKr cells is plotted on the left y-axis. Plasma levels of BNM-III-170 (red) are plotted on the right y-axis. (**B**) Association between plasma levels of BNM-III-170 and plasma binding to CH058TF-infected CEM.NKr cells for timepoints from each respective BNM-III-170 treatment cycle (Spearman correlation).

Plasma concentrations of BNM-III-170 in SHIV-infected animals were measured via liquid chromatography and mass spectrometry at several timepoints following each BNM-III-170 dose for all treatment cycles. Notably, due to blood draw volume constraints, not all early timepoints (0.5, 1, and 1.5 h) were collected for every treatment cycle, limiting our ability to capture and directly compare peak BNM-III-170 plasma concentrations across treatment cycles. However, despite these limitations, we can still conclude that plasma concentrations of BNM-III-170 following the first dose of each treatment cycle in SHIV AD8-EO-infected animals reached, at a minimum, concentrations observed in uninfected RMs (infected C_max_: 4.1–12.8 µg/mL vs. uninfected C_max_: 1.9–15.3 µg/mL). In addition, we found that while BNM-III-170 plasma levels peaked within the first 1.5 h of the first dose of each treatment cycle, they generally remained above the estimated effective concentration of 0.675 µg/mL^37^ 6 h after each dose for all treatment cycles (Fig. 5A).

To assess the ability of bioavailable BNM-III-170 to induce conformational changes in HIV-1 Env, we assessed the capacity of the Abs naturally elicited in these infected RMs to recognize HIV-1-infected cells before and after BNM-III-170 administration. Plasma from treated animals was incubated with CH058TF-infected CEM.NKr cells, and the ability to recognize infected cells was measured by flow cytometry. While RM plasma typically failed to recognize HIV-1-infected cells before BNM-III-170 administration, it efficiently recognized infected cells up to 6 h post-administration depending on the dose used. Importantly, as observed in uninfected RMs, plasma levels of BNM-III-170 in treated, SHIV-infected RMs closely mirrored the kinetic curves for plasma binding. In addition, BNM-III-170 plasma concentrations correlated with plasma binding to infected cells for each treatment cycle (Fig. 5A and B).

**Fig 6:**
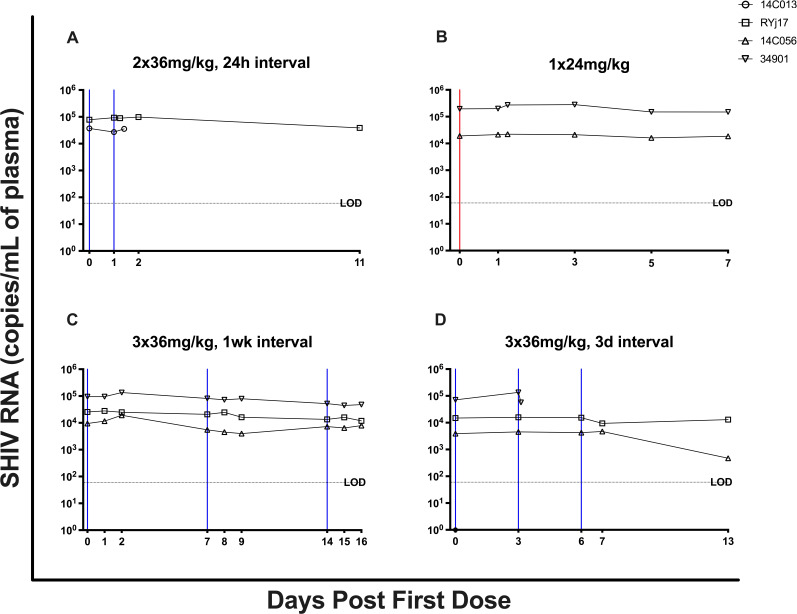
Plasma viral loads for each BNM-III-170 treatment cycle. Plasma viral loads (SHIV RNA copies/mL) were measured by RT-qPCR at multiple timepoints during each BNM-III-170 dosing regimen. The dashed horizontal line represents the assay’s limit of detection (LOD; 60 copies/mL). The red and blue vertical lines represent 36 mg/kg and 24 mg/kg doses of BNM-III-170, respectively.

Next, we evaluated the ability of BNM-III-170 to reduce viral replication in these animals, measuring plasma viral loads (PVLs) at multiple timepoints during and immediately following each BNM-III-170 treatment cycle. PVLs declined by 0.92 logs in animal 14C056 following the 3 × 36 mg/kg, 3-day interval treatment cycle (between 0 h of the first dose and 9 days post third dose). However, no similar changes in PVLs were observed in the three other animals (14C013, RYj17, or 34901) following BNM-III-170 treatments (Fig. 6; Fig. S4). Importantly, given the small number of animals and absence of untreated controls, it is difficult to determine if the observed reduction in PVLs in animal 14C056 was due to BNM-III-170 treatment or to the natural course of SHIV AD8 EO-infection. This highlights the need for additional, larger studies that include an untreated control arm to more accurately determine the virologic impact of BNM-III-170 on SHIV *in vivo*.

**Fig 7:**
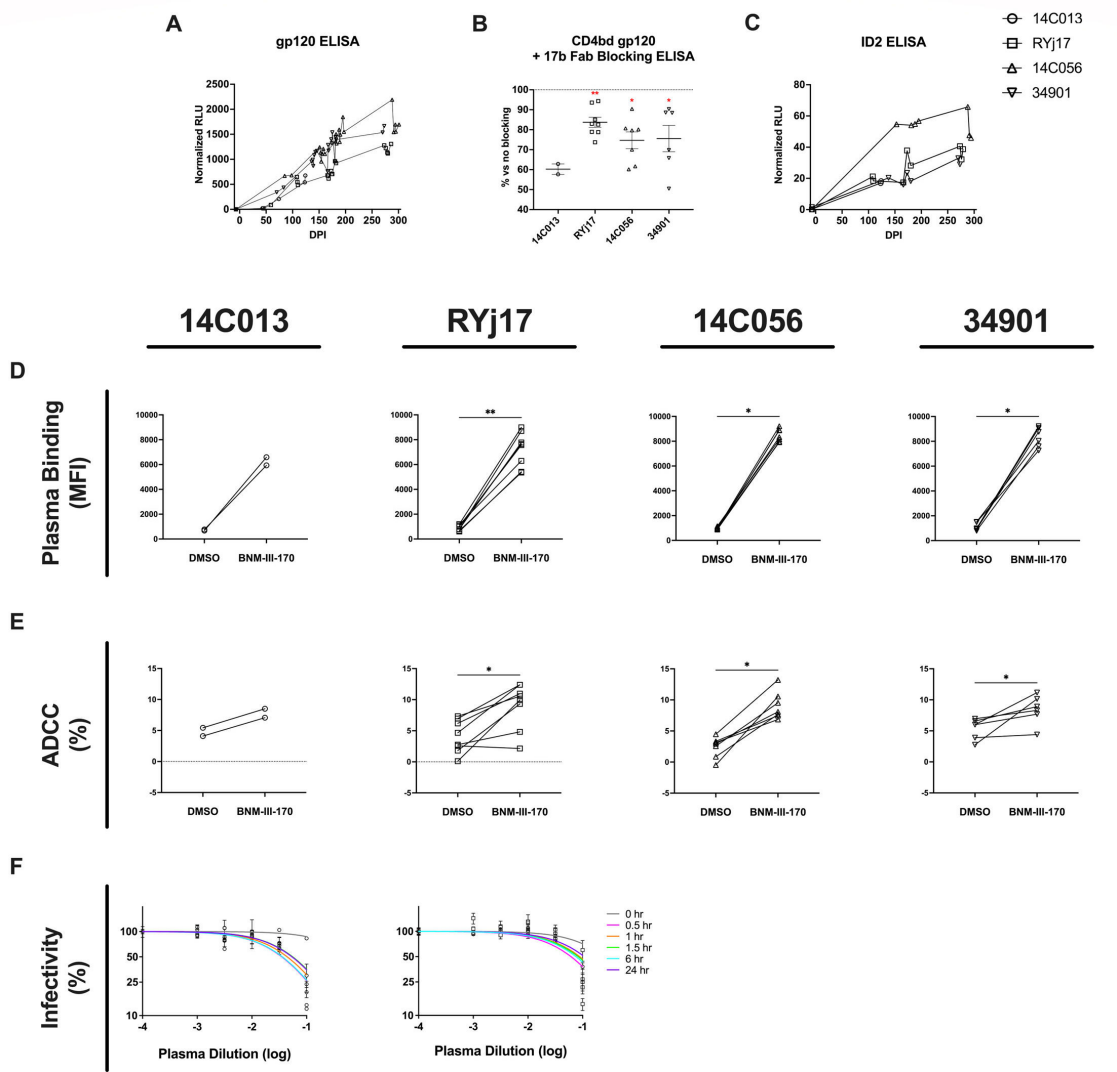
SHIV AD8-EO-infected RMs generate CD4i antibodies capable of binding to infected cells, mediating ADCC, and neutralizing virus following BNM-III-170 treatment. (**A**) Longitudinal CD4i antibody titers were measured via CD4-bound gp120core ELISA for all four animals. (**B**) Percent reduction in plasma binding to CD4 bound gp120core following 17b Fab blocking compared to no blocking. (**C**) Longitudinal anti-cluster A antibody titers were measured via stabilized gp120 inner domain protein (ID2) ELISA for all four animals. (**D**) Capacity of plasma from each animal collected at 0 h of each dose of BNM-III-170 to bind CH058TF-infected CEM.NKr cells in the presence or absence of 50 µM added BNM-III-170. (**E**) Capacity of plasma from each animal collected at 0 h of each dose of BNM-III-170 to mediate ADCC responses against CH58TF-infected CEM.NKr cells in the presence or the absence of 50 µM added BNM-III-170. Statistical analyses for D and E were performed using Wilcoxon matched-pairs signed rank tests. *P-value < 0.05, ** *P*-value < 0.01, ***P-value < 0.001, ****P-value < 0.0001. (**F**) Percent infectivity of SHIV AD8-EO following incubation with indicated dilutions of plasma collected from pre- and post-treatment timepoints during the 2 × 36 mg/kg BNM-III-170 treatment cycle with 1 day dosing interval.

The lack of impact of BNM-III-170 treatment on PVLs in the other three animals could indicate that more sustained levels of BNM-III-170 were needed to significantly impact the number of infected cells. Another possibility is that there were insufficient titers of CD4i-targeted, ADCC-mediating Abs present during BNM-III-170 treatment. While BNM-III-170 is capable of inducing a partial opening of HIV Env to expose the CoRBS, subsequent binding of anti-CoRBS Abs to Env is required for the exposure of the highly conserved, gp120 Constant region 1 and 2 (C1-C2) epitope targeted by anti-cluster A Abs that mediate ADCC (26, 39). Therefore, if anti-CoRBS or anti-cluster A Abs were not present at sufficient concentrations at the time of BNM-III-170 treatment, we would expect no BNM-III-170-stimulated increase in ADCC-mediated clearance of infected cells. While CD4i Abs including anti-CoRBS and anti-cluster A Abs have been detected as early as 3 months post-infection in PWH (15), ADCC activity has been observed as early as 1 month post-infection (16). However, whether and when anti-CoRBS and anti-cluster A Abs are elicited during SHIV AD8-EO infection is unclear. To confirm the presence of CD4i Abs in the plasma of SHIV AD8-EO-infected RMs, we performed ELISAs on longitudinal plasma samples using the CD4-bound gp120core in which both anti-CoRBS and anti-cluster A Env epitopes are exposed. CD4i Abs were consistently detected in the plasma of all four animals within the first few months of SHIV AD8-EO infection, with titers continuing to increase throughout the course of the study (Fig. 7A). To confirm that anti-CoRBS Abs were present in these animals at the time of BNM-III-170 treatment, we next performed competition ELISAs with the CD4-bound gp120core ± 17 b Fab on plasma collected at 0 h of each BNM-III-170 dose for all treatment cycles. 17b Fab blocking was shown to result in a reduction of plasma binding to the CD4-bound gp120core, indicating that anti-CoRBS Abs were elicited in SHIV AD8-EO-infected RMs and were present at the time of BNM-III-170 administration (Fig. 7B). Next, to determine whether anti-cluster A Abs were elicited during SHIV AD8-EO infection, we performed ELISAs with the stabilized gp120 inner domain protein ID2 which expresses the epitope of the anti-cluster A mAb A32 (21). ID2-reactive Abs were detected in all four SHIV AD8-EO-infected RMs, indicating that anti-cluster A Abs were also elicited in these animals (Fig. 7C). Of note, the highest levels of both CD4i and anti-cluster A Abs were observed in animal 14C056 at 0 h of 288 dpi, immediately prior to the 3 × 36 mg/kg, 3 day interval BNM-III-170 dosing regimen which resulted in the 0.92 log reduction in PVLs. However, even the CD4i and ID2-reactive Ab titers in this animal were demonstrated to be lower than those elicited in PWH at similar post-infection timepoints (Fig. S7A and B).

After confirming that the anti-CoRBS and anti-cluster A Abs were present in the plasma of SHIV AD8-EO-infected RMs, we next assessed the ability of the Abs in the plasma of these infected animals to (i) bind HIV Env, (ii) mediate ADCC, and (iii) neutralize SHIV AD8 in the presence of BNM-III-170. As noted previously, we saw increased plasma binding to CH058TF-infected CEM.NKr cells with increasing levels of bioavailable BNM-III-170 in the plasma of treated SHIV AD8-EO-infected animals (Fig. 5A and B). Here, we also compared the ability of plasma collected from 0 h of each BNM-III-170 dose to recognize and eliminate CH058TF-infected CEM.NKr cells by ADCC in the absence vs. presence of a set amount (50 μM) of added BNM-III-170. We found that BNM-III-170 significantly increased the binding and ADCC activity of Abs present in 0 h plasma from each animal against infected cells (Fig. 7D and E). Finally, we evaluated the ability of plasma collected before and during the 2 × 36 mg/kg, 1 day interval BNM-III-170 treatment cycle to neutralize SHIV AD8-EO using the TZM-bl neutralization assay. Plasma collected immediately prior to the first dose of BNM-III-170 in this treatment cycle exhibited little neutralization of SHIV-AD8; however, SHIV AD8-EO was neutralized by RM plasma obtained at several timepoints after BNM-III-170 administration (Fig. 7F). Importantly, neutralization was observed with plasma obtained 24 h after BNM-III-170 administration, a time when BNM-III-170 levels in the plasma were low; this argues against a direct antiviral effect of BNM-III-170 in this assay and supports a model where BNM-III-170 sensitizes SHIV AD8-EO virions to neutralization by otherwise non-neutralizing Abs.

In summary, the two classes of Abs (anti-CoRBS and anti-cluster A Abs) that have previously been shown to mediate ADCC in the presence of BNM-III-170 were detected in SHIV AD8-EO-infected RMs in this study. In addition, Abs in the plasma of these infected RMs were shown to be capable of (i) binding to HIV-infected cells, (ii) mediating ADCC, and (iii) neutralizing virus in the presence of BNM-III-170. However, in spite of this, no consistent decreases in plasma viral loads were observed in viremic SHIV AD8-EO-infected RMs following BNM-III-170 dosing. Higher levels of anti-CoRBS and anti-cluster A Abs may be required for BNM-III-170 to impact viral loads *in vivo,* particularly in a setting with large numbers of infected cells such as the viremic animals in our study.

## DISCUSSION

Due to the persistence of replication-competent HIV-1 in a reservoir of infected CD4+ T cells, cessation of ART results in viral rebound in the large majority of PWH. Therefore, lifelong adherence to ART is required to maintain viral suppression (2–8). To eliminate the need for lifelong ART, significant efforts over the past three decades have focused on designing interventions aimed at achieving an HIV-1 cure (40).

Early in the HIV epidemic, the ability of recombinant soluble CD4 (sCD4) to reduce HIV infectivity was evaluated. While sCD4 inhibited the binding of HIV to CD4+ lymphocytes and reduced the infectivity of laboratory strains of HIV *in vitro*, (41) sCD4 administration in PWH was observed to have no significant impact on viral loads across multiple clinical studies (42, 43). The resistance of most primary HIV-1 Envs to sCD4 engagement may explain why sCD4 demonstrated only minimal *in vivo* potential as an antiviral agent (44). While cells infected with lab-adapted HIV-1 viruses are sensitive to sCD4-induced conformational changes and subsequent ADCC sensitization, cells infected with primary HIV-1 isolates are largely resistant (16, 26). This resistance is likely due to the quaternary architecture of primary Envs, which limits engagement by larger proteins like sCD4 (~55,000 Da) and mini-protein CD4 mimics like M48U1 (~3,000 Da). Small-molecule CD4mcs are comparatively smaller at ~700 Da, likely enabling them to bypass these structural constraints (45). Compared to sCD4 and M48U1, CD4mcs exhibit significantly improved responses in sensitizing cells infected with primary HIV-1 isolates to ADCC (16).

CD4mc can also inhibit HIV-1 infection more effectively than sCD4 by prematurely activating Env, leading to irreversible inactivation, and was found to be significantly more potent against a broad range of HIV-1 viruses than sCD4 (45).

BNM-III-170 is a CD4mc that has been shown to significantly delay viral rebound when administered in combination with anti-CoRBS and anti-cluster A Abs at ART interruption in HIV-1-infected humanized mice (27). Here, we characterized the safety, pharmacokinetics, and biological activity of BNM-III-170 in uninfected and SHIV AD8-EO-infected RMs to inform future, larger studies evaluating the ability of BNM-III-170 to reduce the viral reservoir.

Our study established several BNM-III-170 dosing regimens that were well-tolerated in SHIV-infected RMs. While adverse events of unknown cause and potential liver injury were observed in one infected animal that received daily 36 mg/kg doses of BNM-III-170, no major concerns were noted with all other evaluated BNM-III-170 dosing regimens, including 36 mg/kg administered every 3 days. However, transient elevations in liver enzymes, WBCs, neutrophils, and monocytes as well as transient, non-specific lymphopenia were consistently observed following BNM-III-170 dosing, highlighting the need for continued monitoring of blood chemistries and CBCs in future BNM-III-170 *in vivo* studies. Pharmacokinetic analysis of plasma BNM-III-170 demonstrated that BNM-III-170 has a relatively short half-life in both uninfected and SHIV-infected RMs, suggesting that repeat dosing may be necessary to maintain therapeutic levels. Taken together, the results of our study indicate that 36 mg/kg doses of BNM-III-170 administered once every 3 days have the most favorable benefit-to-risk profile of the evaluated BNM-III-170 dosing regimens.

Importantly, we showed that the plasma of treated animals not only displayed enhanced binding to infected cells but also neutralization of SHIV AD8-EO virions. Of note, plasma collected 24 h after BNM-III-170 administration still neutralized viral particles, supporting earlier findings that sub-inhibitory concentrations of CD4mc sensitize infectious viral particles to neutralization by otherwise non-neutralizing Abs (28, 46, 47). This effect was shown to depend on CD4mc-induced conformational changes in HIV Env, which expose epitopes targeted by non-neutralizing Abs, allowing these Abs to bind and block viral entry. We also show that SHIV AD8-EO-infected RMs, similar to PWH, naturally elicit Abs during infection that are capable of mediating ADCC in the presence of BNM-III-170. Together, these results highlight the potential for small CD4-mimetics to target cell-free viruses through direct neutralization and promote the elimination of infected cells.

In spite of the demonstrated biological activity of BNM-III-170 in the plasma of SHIV-infected RMs, no consistent decreases in PVLs were observed with BNM-III-170 dosing; this is in contrast to what was observed in Rajashekar et al. where BNM-III-170 coadministration with anti-CoRBS and anti-cluster A Abs or PWH plasma (i) reduced plasma viral loads and CD4+ T cell-associated proviral DNA in viremic humanized mice and (ii) delayed viral rebound after ATI in ART-treated mice as compared to their respective mock-treated control groups (27).

Several factors may explain the disparate results observed in HIV-infected humanized mice vs. SHIV-infected RMs. These include differences in (i) BNM-III-170 dosing regimens, (ii) anti-CoRBS and anti-cluster A Ab levels, and (iii) NK cell functionality. In HIV-infected humanized mice, BNM-III-170 was administered at a dose of 3 mg/day, which is roughly equivalent to 100 mg/kg daily; this dosing regimen led to sustained BNM-III-170 plasma concentrations above 200 ng/mL during the treatment cycle. Conversely, in SHIV-infected RMs, the highest administered BNM-III-170 dose was 36 mg/kg and the shortest tolerated dosing frequency was once every 3 days, resulting in plasma BNM-III-170 declining to levels below the limit of detection between doses. Given the short half-life of BNM-III-170 and observed tolerability issues, it may be beneficial to evaluate if newer CD4mcs with higher potency such as indoline CD4mcs are better tolerated and capable of reducing the SHIV reservoir in RMs (37, 45). Another difference between the mouse and RM studies was that the mice passively received infusions of both anti-CoRBS and anti-cluster A Abs, whereas our study relied on the natural elicitation of these Abs during SHIV infection in RMs. As mentioned previously, BNM-III-170 only leads to a partial opening of the HIV-1 Env to expose CoRBS, and anti-CoRBS Abs are necessary to fully push Env into an “open” conformation where anti-cluster A Abs can bind and mediate ADCC (26, 39).

In our study, we show that CD4i Abs including anti-CoRBS and anti-cluster A Abs are elicited naturally during SHIV AD8-EO infection; however, these titers were lower than those elicited in PWH at similar post-infection timepoints. Although it was previously demonstrated that depletion of CD20+ B cells in SIV-infected RMs did not impact plasma viral load (44), it is possible that lower levels of anti-CoRBS and anti-cluster A Abs may limit the ability of BNM-III-170 to reduce the viral reservoir. Therefore, future studies in SHIV-infected RMs should explore the possibility of co-administering anti-CoRBS and anti-cluster A Abs in combination with BNM-III-170. Similarly, CD4mc-based interventions may be more beneficial if performed when there are a lower number of infected cells to target and eliminate, for example at ART initiation or ART interruption. Finally, another factor that may explain the variable impact of BNM-III-170 in viremic mice vs. RMs is NK cell function. The mice used in Rajashekar et al. were humanized SRG-15 mice which were generated through knocking in human interleukin 15 (*IL15*) and human signal regulatory protein alpha (*SIRPA*). Importantly, SRG-15 mice have previously been shown to support the development of functional ADCC-mediating cells, including NK cells (48). In our study, effector cells for ADCC were generated in the background of SHIV infection, with potential perturbation of the immune system. Future studies may consider combinatorial approaches with IL-15 or IL-21 therapy to enhance NK-cell function (49–51).

In summary, we developed BNM-III-170 dosing regimens in SHIV-infected RMs that were well tolerated and demonstrated that bioavailable BNM-III-170 in the plasma of treated animals was capable of increasing plasma binding to infected cells and neutralization of autologous virus. This study is the first to evaluate the therapeutic efficacy of BNM-IIII-170 in chronically SHIV-infected RMs and establishes an important foundation for understanding the pharmacokinetics of BNM-III170 and its biological activity. While BNM-III-170 dosing did not have an impact on PVLs of SHIV-infected RMs in this study, future, larger studies in viremic and ART-treated RMs are necessary to fully determine the virologic impact of BNM-III-170 and other CD4mcs.

## MATERIALS AND METHODS

Portions of the materials and methods have been previously reported (52–56) and are summarized below.

### Study approval

The uninfected rhesus macaque (RM) study was performed at the Wisconsin National Primate Research Center (WNPRC). The uninfected RMs used for the BNM-III-170 dose-escalation study were housed at WNPRC in accordance with the standards of the Association for the Assessment and Accreditation of Laboratory Animal Care (AAALAC) and the University of Wisconsin Research Animal Resources Center and Compliance unit (UWRARC). Animal experiments were approved by the University of Wisconsin College of Letters and Sciences and the Vice Chancellor for Research and Graduate Education Centers AICUC (protocol numbers G005609 and G006679) and performed in compliance with the principles described in the *Guide for the Care and Use of Laboratory Animals* (57). Fresh water was always available, commercial monkey chow was provided twice a day, and fresh produce was supplied daily. To minimize any pain and distress related to experimental procedures, ketamine HCL was used to sedate animals prior to blood collection, and animals were monitored twice a day by animal care and veterinary staff. The animals were socially housed in pairs or groups of compatible animals whenever possible.

The SHIV-infected RM study was performed at EPC. EPC’s animal care facilities are accredited by both the U.S. Department of Agriculture (USDA) and the Association for Assessment and Accreditation of Laboratory Animal Care (AAALAC). All animal procedures were performed in line with institutional regulations and guidelines set forth by the NIH’s Guide for the Care and Use of Laboratory Animals, 8th edition, and were conducted under anesthesia with appropriate follow-up pain management to minimize animal suffering. All animal experimentation was reviewed and approved by Emory University’s Institutional Animal Care and Use Committee (IACUC) under permit PROTO201700553.

### Animal models

For the uninfected RM study, 2 female and 3 male (average age of 4 years and 6 months) Indian-origin RMs (*Macaca mulatta*) were housed at WNPRC. For the SHIV-infected RM study, 4 female (average age of 4 years and 6 months) specific pathogen-free (SPF) *Mamu*-A*01^–^, B*08^–^, B*17^–^ Indian-origin RMs (*Macaca mulatta*) were housed at EPC in the BSL-2 facility as previously described (6). All 4 RMs were infected intravenously with 200 TCID_50_ SHIV AD8-EO generously provided by Malcolm Martin.

### BNM-III-170 synthesis

BNM-III-170 bis-trifluoroacetic acid (TFA) salt was synthesized as previously described (14).

### BNM-III-170 administration

BNM-III-170 TFA salt was dissolved in dimethyl sulfoxide (DMSO; STEMSOL Cat. No. PP1300) to yield a 100 mM BNM-III-170 TFA salt solution. The 100 mM BNM-III-170 salt solution was further diluted in phosphate-buffered saline (PBS; RMBI PBS: REF# BSS-PBS-1 × 6) to yield a 20% DMSO, 13.5 mg/mL BNM-III-170 TFA salt solution. This solution was then filtered through a 0.2 μm DMSO-safe luer lock filter (Acrodisc DMSO-safe 0.2 μm nylon syringe filter, REF: 4433). In the SHIV-infected RM study, additional filtration steps were added prior to and following dilution in PBS, for a total of 3 filtrations.

In the uninfected RM study, BNM-III-170 was administered at 3–36 mg/kg doses. One RM was administered a 3 mg/kg dose of BNM-III-170 intravenously. All other doses were administered subcutaneously on the back between the shoulder blades so that the animals could not scratch at the site of injection.

For the SHIV-infected RM study, RMs were pre-treated with diphenhydramine (1 mg/kg IV) prior to receiving BNM-III-170. BNM-III-170 was administered at doses of 24 mg/kg or 36 mg/kg subcutaneously to RMs. Given the large volume required to reach these doses, the solution was divided between three injection sites in either the right shoulder, left shoulder, or upper back regions. To monitor injection site-specific reactions, these regions were rotated between each administration cycle. Animals also received the antiemetic Cerenia (1 mg/kg SQ) for potential treatment-related nausea following BNM-III-170 administration.

### Sample collection and processing

Plasma, peripheral blood mononuclear cells (PBMCs), and lymph node (LN) biopsies were collected longitudinally and processed as previously described (6, 58). CBCs and comprehensive serum blood chemistries were performed routinely to monitor animal health and determine absolute cell counts.

### Immunophenotyping

A 20-parameter flow cytometric analysis was performed on fresh PBMCs and mononuclear cells (10^6^ cells) derived from LN biopsies using anti-human monoclonal Abs (mAbs), which we (6, 58–60) and others, including databases maintained by the NHP Reagent Resource (MassBiologics), have shown as being cross-reactive in RMs. A panel of the following mAbs was used for phenotyping in PBMCs and LNs: anti-CD3-BUV395 (clone SP34-2; cat. #564117), anti-CD8-BUV496 (clone RPA-T8; cat. 564804), anti-CD28-BUV737 (clone CD28.2; cat. # 564438), anti-Ki-67-BUV480 (clone B56; cat. # 566172), anti-CD56-BV711 (clone B159; cat. #740781), anti-CCR2-BB700 (clone 48607; cat #564067), anti-CCR7-BB700 (clone 3D12; cat. #566437), anti-CD14-Cy7PE (clone M5E2; cat. #557742), anti-CD16-BUV805 (clone 3G8; cat. #748850), and anti-CD20-BUV563 (clone 2H7, cat. #748456) from BD Biosciences; anti-CD4-APC/Cy7 (clone OKT4; cat. #317418), anti-HLA-DR-BV560 (clone L243; cat. #307650), anti-PD-1-BV785 (clone EH12.2H7; cat. #329930), and anti-CD95-BV605 (clone DX2; cat. #305628) from Biolegend; anti-CXCR5-PE (clone MU5UBEE; cat. #12–9185-42) from eBioscience; anti-CD27-PECy5 (clone 1A4CD27; cat. #6607107) and anti-NKG2a-APC (clone Z199; cat. #A60797) from Beckman Coulter; anti-GranB-PETR (clone GB11; cat. #GRB17) from eBioscience; and anti-IgD-FITC (polyclonal; cat. #2030–02) from Southern Biotech. mAbs for chemokine receptors (i.e., CCR2, CCR7) were incubated at 37°C for 15 min, and cells were fixed and permeabilized for 30 min at room temperature using a FoxP3/Transcription Factor Staining Buffer Kit (Tonbo Biosciences; cat. # TNB-0607-KIT). All samples were fixed with 1% paraformaldehyde and acquired within 24 h of fixation. Acquisition of data was performed on a FACSymphony A5 (BD Biosciences) driven by FACS DiVa software and analyzed with FlowJo (version 10.7; Becton, Dickinson, and Company).

### Viral loads

Plasma SHIV viral loads (SHIV-RNA copies/mL) were determined as previously described (61) using quantitative reverse transcription PCR (RT-qPCR) assays with a limit of detection of 60 copies/mL.

### Cell lines and primary cells

293T human embryonic kidney cells (obtained from ATCC), HeLa TZM-bl cells (obtained from the NIH AIDS reagent program), and CEM.NKr-CCR5-sLTR-Luc cells were grown as previously reported (16, 52). Primary human PBMCs were isolated by leukapheresis and cultured in RPMI 1640 (Thermo Fisher Scientific, Waltham, MA, USA) complete medium as previously described (16). Briefly, PBMCs were obtained by leukapheresis, and CD4+ T lymphocytes were from resting PBMCs by negative selection using immunomagnetic beads per the manufacturer’s instructions (StemCell Technologies, Vancouver, BC) and were activated with phytohemagglutinin-L (10 µg/mL) for 48 h and then maintained in RPMI 1640 (Thermo Fisher Scientific, Waltham, MA, USA) complete medium supplemented with rIL-2 (100 U/mL).

### Plasmids and proviral constructs

Transmitted/Founder (T/F) infectious molecular clone (IMC) of patient CH058 was previously described (62). The vesicular stomatitis virus G (VSV-G)-encoding plasmid was previously described (63). The SHIV-AD8-EO, SHIV-C5, and SHIV-CH040 IMC were previously described (64–66).

### Viral production and infections

For *in vitro* infection, vesicular stomatitis virus G (VSV-G)-pseudotyped HIV-1/SHIV viruses were produced by co-transfection of 293T cells with an HIV-1/SHIV proviral construct and a VSV-G-encoding vector using the calcium phosphate method. Two days post-transfection, cell supernatants were harvested, clarified by low-speed centrifugation (300 × *g* for 5 min), and concentrated by ultracentrifugation at 4°C (100,605 × *g* for 1 h) over a 20% sucrose cushion. Pellets were resuspended in fresh RPMI, and aliquots were stored at −80°C until use. Viral preparations were titrated directly on primary CD4+ T cells and CEM.NKr cells to achieve similar levels of infection. Viruses were then used to infect activated primary CD4+ T cells from healthy HIV-1-negative donors or CEM.NKr cells by spin infection at 800 × *g* for 1 h in 96-well plates at 25°C. All experiments using VSV-G-pseudotyped HIV-1 isolates were done in a biosafety level 3 (BSL3) laboratory following manipulation protocols accepted by the CRCHUM Biosafety Committee, which respects the requirements of the Public Health Agency of Canada.

### Antibodies and human plasma

The anti-CoRBS mAbs 17b was conjugated with Alexa-Fluor 647 probe (Thermo Fisher Scientific) as per the manufacturer’s protocol and used for cell-surface staining of HIV-1-infected cells in the presence of plasma from RM. Unconjugated 17b and the anti-V3 crown 19b mAbs (NIH AIDS Reagents program) were used in neutralization and flow cytometry experiments. Goat anti-human IgG Alexa-Fluor 647 secondary Ab (Thermo Fisher Scientific) was used to determine overall unconjugated antibody and plasma binding. Plasma from PWH, obtained from the FRQS-AIDS and Infectious Diseases Network (the Montreal Primary HIV Infection Cohort (67, 68), was collected, heat-inactivated, and conserved as previously described (16).

### Antibody production

FreeStyle 293F cells (Thermo Fisher Scientific) were grown in FreeStyle 293F medium (Thermo Fisher Scientific) to a density of 1  ×  10^6^ cells/mL at 37°C with 8% CO_2_ with regular agitation (150 rpm). Cells were transfected with plasmids expressing the light and heavy chains of 17b (kindly provided by James Robinson) using ExpiFectamine 293 transfection reagent, as directed by the manufacturer (Thermo Fisher Scientific). One week later, the cells were pelleted and discarded. The supernatants were filtered (0.22-μm-pore-size filter), and antibodies were purified by protein A affinity columns, as directed by the manufacturer (Cytiva, Marlborough, MA, USA). 17b Fab fragments were prepared from purified IgG (10  mg/mL) by proteolytic digestion with immobilized papain (Pierce, Rockford, IL) and purified using protein A, followed by gel filtration chromatography on a Superdex 200 16/60 column (Cytiva).

### Flow cytometry analysis of cell-surface staining

Cell surface staining was performed at 48 h post-infection. Mock-infected or HIV-1-infected CEM.NKr or primary CD4+ T cells were incubated for 45 min at 37°C with anti-Env mAbs (5 µg/mL), plasma from PWH (1:1,000 dilution), or plasma from infected RMs (1:500). When indicated, antibody/plasma incubation was done in the presence of 50 µM of BNM-III-170, or equivalent amounts of DMSO. To evaluate the capacity of plasma from SHIV-AD8-EO-infected RMs treated with BNM-III-170 to recognize HIV-1-infected cells, a 1:10 plasma dilution was used. Cells were then washed twice with PBS and stained with the appropriate Alexa Fluor 647-conjugated secondary antibody (2 µg/mL) for 20 min and the viability dye AquaVivid (Thermo Fisher Scientific) at room temperature. Cells were then washed twice with PBS and fixed in a 2% PBS-formaldehyde solution. Infected cells were then permeabilized using the Cytofix/Cytoperm Fixation/Permeabilization Kit (BD Biosciences, Mississauga, ON, Canada) and stained intracellularly using PE or FITC-conjugated mouse anti-p24 mAb (clone KC57; Beckman Coulter, Brea, CA, USA; 1:100 dilution) for HIV-1 infection or using Alexa Fluor 488-conjugated mouse anti-p27 mAb (clone 2F12) for SHIV infection. Samples were acquired on an LSR II cytometer (BD Biosciences), and data analysis was performed using FlowJo v10.5.3 (Tree Star, Ashland, OR, USA).

To evaluate the functional activity of bioavailable BNM-III-170 in plasma, the plasma from uninfected RMs treated with BNM-III-170 was used to modulate Env conformation at the surface of infected cells. Briefly, 2 days post-infection, CEM.NKr cells infected with CH058TF were incubated with plasma from RMs (1:10 dilution), the Alexa-Fluor 647-conjugated 17b (5 µg/mL), and the AquaVivid viability dye at 37°C for 45 min. As a positive control, infected cells were incubated with 50 µM of BNM-III-170. Infected cells were then identified based on HIV-1 p24 detection as described above.

### FACS-based ADCC assay

Measurement of ADCC using the FACS-based assay was performed at 48 h post-infection, as previously described. Briefly, CEM.NKr or primary CD4+ T cells infected with indicated HIV-1 or SHIV IMCs were stained with AquaVivid viability dye and cell proliferation dye (eFluor670; eBioscience) and used as target cells. Human PBMC effector cells, stained with another cellular marker (cell proliferation dye eFluor450; eBioscience), were added at an effector:target ratio of 10:1 in 96-well V-bottom plates (Corning). Plasma from RMs (1:500 dilution) or PWH (1:1,000 dilution) with 50 µM of BNM-III-170, or equivalent amounts of DMSO, was added to appropriate wells, and cells were incubated for 15 min at room temperature. The plates were subsequently centrifuged for 1 min at 300 × *g*, and incubated at 37°C, 5% CO_2_ for 4–5 h before being fixed in a 2% PBS-formaldehyde solution. Infected cells were identified by anti-p24 or anti-p27 intracellular staining as described above. Samples were acquired on an LSR II cytometer (BD Biosciences), and data analysis was performed using FlowJo v10.5.3 (Tree Star, Ashland, OR, USA). The percentage of ADCC was calculated with the following formula: (% of p24+/p27+ cells in Targets plus Effectors) − (% of p24+/p27+ cells in Targets plus Effectors plus Abs) / (% of p24+/p27+ cells in Targets) by gating on infected live target cells.

### Luciferase ADCC assay

Measurement of ADCC responses using the Luciferase assay was performed as previously described (53). Briefly, CEM.NKR-CCR5-sLTR-Luc target cells, which express firefly luciferase (Luc) under the control of a Tat-inducible promoter, were infected and used as target cells 48 h post-infection with indicated SHIV IMCs. Primary human PBMCs were used as effector cells. Target and effector cells were co-cultured for 6–8 h, in triplicate, at an effector:target ratio of 10:1 in the presence of PWH plasma. The loss of Luc activity was measured as an indication of plasma-mediated killing of productively infected cells. Infected target cells incubated with effector cells in the absence of plasma were used to measure maximal Luc activity, and uninfected target cells cultured with effector cells were used to determine background Luc activity. The percentage of ADCC was calculated with the following formula: [(relative reading of Luc in targets plus effectors) − (relative reading of Luc in targets plus effectors plus Abs or sera)] / (relative reading of Luc in targets alone).

### Measurement of BNM-III-170 in rhesus macaque plasma by mass spectrometry

BNM-III-170 levels in plasma from rhesus macaque were determined by high-performance liquid chromatography-tandem mass spectrometry (HPLC-MS/MS). Briefly, BNM-III-170 was extracted from plasma using protein precipitation. A Thermo Scientific TSQ Quantiva triple quadrupole mass spectrometer was interfaced with the Thermo Scientific Ultimate 3000XRS UHPLC system using a heated electrospray ion source (HESI). MS detection was performed in positive ion mode, using multiple reaction monitoring (MRM). The precursor-ion reactions for BNM-III-170 and IS were set to 447.3 → [226.1 + 357.1] and 262.0 → 139.1, respectively. Chromatographic separation was achieved on a Thermo Scientific Accucore RP-MS analytical column (100 × 2.1 mm I.D., 2.6 mm) maintained at 40°C using isocratic elution, which consisted of acetonitrile and 10 mM ammonium formate in water (pH 3.0) at a ratio of 20:80, respectively. The flow rate was fixed at 300 µL/min. Acquisition and analysis of data were conducted using the Xcalibur 4.0 software. Each calibration curve was comprised of a double blank (matrix without analyte or internal standard), zero standard, and eight calibration points. Calibration curves were computed from the equation y = ax + b, based on the weight of the calibration lines created from the peak-area ratios of the drug relative to the internal standard.

### Production of recombinant gp120

The recombinant CD4-bound stabilized gp120core (ΔV1V2V3V5) and the inner domain stabilized (ID2) gp120 proteins were produced and purified as previously reported (54, 55).

### Enzyme-linked immunosorbent assays

Bovine serum albumin (BSA), CD4-bound stabilized gp120core (CD4bd gp120core), and stabilized gp120 inner domain (ID2) were prepared in PBS (0.1 µg/mL) and adsorbed to MaxiSorp; Nunc plates (Thermo Fisher Scientific, Waltham, MA, USA) overnight at 4°C. BSA was used as a negative control. Coated wells were subsequently blocked with blocking buffer (Tris-buffered saline (TBS) containing 0.1% Tween 20 and 2% [wt/vol] BSA) for 60 min at room temperature. Wells were then washed four times with washing buffer (Tris-buffered saline [TBS] containing 0.1% Tween 20). For Fab blocking experiments, anti-CoRBS 17b Fab fragments (10 µg/mL) were prepared in diluting buffer (Tris-buffered saline (TBS) containing 0.1% Tween 20 and 0.1% [wt/vol] BSA) and incubated with half of the CD4bd gp120core-coated wells for 60 min at room temperature. Wells were then washed four times with washing buffer. Plasma, the anti-cluster a A32 mAb, or the anti-CoRBS 17b Abs were diluted in diluting buffer and incubated for 90 min at room temperature. Wells were then washed four times with washing buffer. This was followed by incubation of horseradish peroxidase (HRP)-conjugated antibody specific for the Fc region of human IgG (0.4 µg/mL; Thermo Fisher Scientific, Waltham, MA, USA) for 60 min at room temperature. Wells were then washed four times with washing buffer. HRP enzyme activity was determined after the addition of a 1:1 mix of Western Lightning ECL reagents (Perkin Elmer Life Sciences, Waltham, MA, USA). Light emission was measured with an LB 941 TriStar luminometer (Berthold Technologies, Bad Wildbad, Germany). The signal obtained with BSA was subtracted for each plasma and was then normalized to the signal obtained with 17b (ELISA with CD4bd gp120core) or A32 (ELISA with ID2) present in each plate.

### Viral neutralization assay

The viral neutralization assay was done as previously described (56). Briefly, TZM-bl target cells were seeded at a density of 1  ×  10^4^ cells/well in 96-well luminometer-compatible tissue culture plates (Perkin-Elmer Life Sciences, Waltham, MA) 24 h before infection. Then, 100 µL portions of recombinant viruses was incubated with the indicated amount of mAbs, plasma, BNM-III-170, or a mixture of mAbs and BNM-III-170 for 1 h at 37°C and then added to the target cells. After incubation for 48 h at 37°C, the medium was removed from each well, and the cells were lysed by the addition of 30 µL of passive lysis buffer (Promega) and three freeze-thaw cycles. An LB 941 TriStar luminometer (Berthold Technologies) was used to measure the luciferase activity in each well after the addition of 100 µL of luciferin buffer (15 mM MgSO_4_, 15 mM KPO_4_ [pH 7.8], 1  mM ATP, and 1 mM dithiothreitol) and 50 µL of 1  mM d-luciferin potassium salt (Prolume).

### Statistical analysis

All statistical analyses were performed two-sided with *P*-values ≤ 0.05 deemed significant. Ranges of significance were graphically annotated as follows: *P < 0.05; **P < 0.01; ***P < 0.001; ****P < 0.0001. Analyses for Fig. 1E, 2C through F, 4A, B, 5B, 7B, D, and E; Fig. S2A through F were performed with Prism version 8 (GraphPad).

## Data Availability

The data that support the findings of this study are available from the corresponding author upon reasonable request.

## SUPPLEMENTAL MATERIAL

Fig. S1 jvi.00062-25-s0001.tiff Enantioselective synthesis of (+)-(R,R,)-BNM-III-170.

Fig. S2 jvi.00062-25-s0002.tiff 17b binding increases with increased concentrations of bioavailable BNM-III-170 in treated, uninfected RMs.

Fig. S3 jvi.00062-25-s0003.jpg Study design by collection group of subcutaneous BNM-III-170 treatment in SHIV AD8-EO-infected RMs.

Fig. S4 jvi.00062-25-s0004.jpg Longitudinal SHIV plasma viral loads for each animal.

Fig. S5 jvi.00062-25-s0005.tiff Longitudinal frequencies and absolute numbers of lymphocytes following the first dose of each treatment cycle of BNM-III-170.

Fig. S6 jvi.00062-25-s0006.tiff Flow cytometry gating strategy.

Fig. S7 jvi.00062-25-s0007.tiff Comparison of antibody titers in the plasma of PLWH and SHIV AD8-EO-infected RMs.

Supplemental legends jvi.00062-25-s0008.docx Legends for Fig. S1 to S7 and Table S1.

Table S1 jvi.00062-25-s0009.xlsx Uninfected and SHIV AD8-EO-infected rhesus macaque characteristics.

ASM does not own the copyrights to Supplemental Material that may be linked to, or accessed through, an article. The authors have granted ASM a non-exclusive, world-wide license to publish the Supplemental Material files. Please contact the corresponding author directly for reuse.
