# Supplementary figures and images for: Safety, pharmacokinetics, and biological activity of CD4-mimetic BNM-III-170 in SHIV-infected rhesus macaques
Source: J Virol. 2025 Apr 7;99(5):e00062-25. doi: 10.1128/jvi.00062-25 (PMC12090809; doi:10.1128/jvi.00062-25)

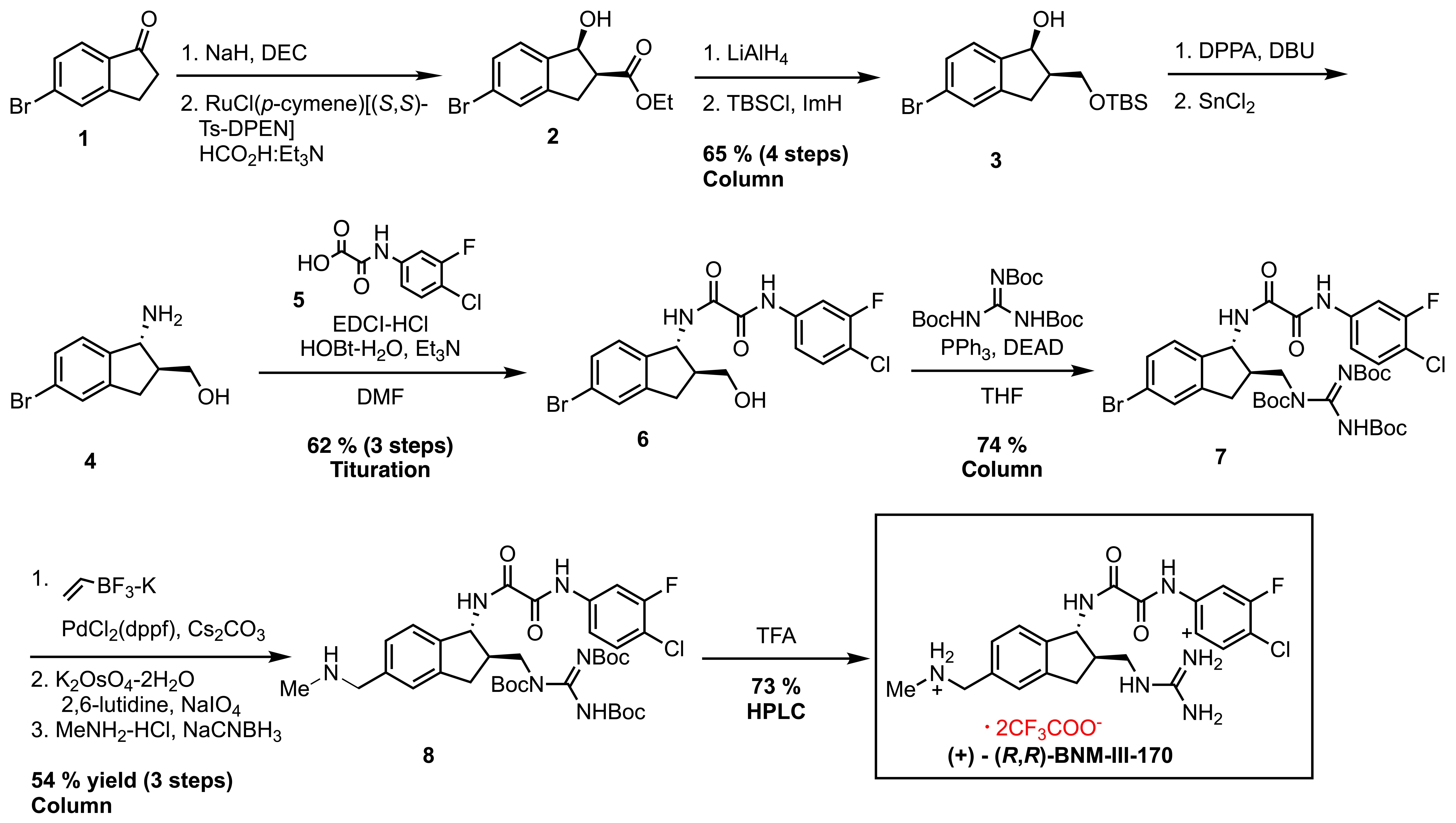

Supplement: Fig. S1 — Enantioselective synthesis of (+)-(R,R,)-BNM-III-170. [file jvi.00062-25-s0001.tiff]

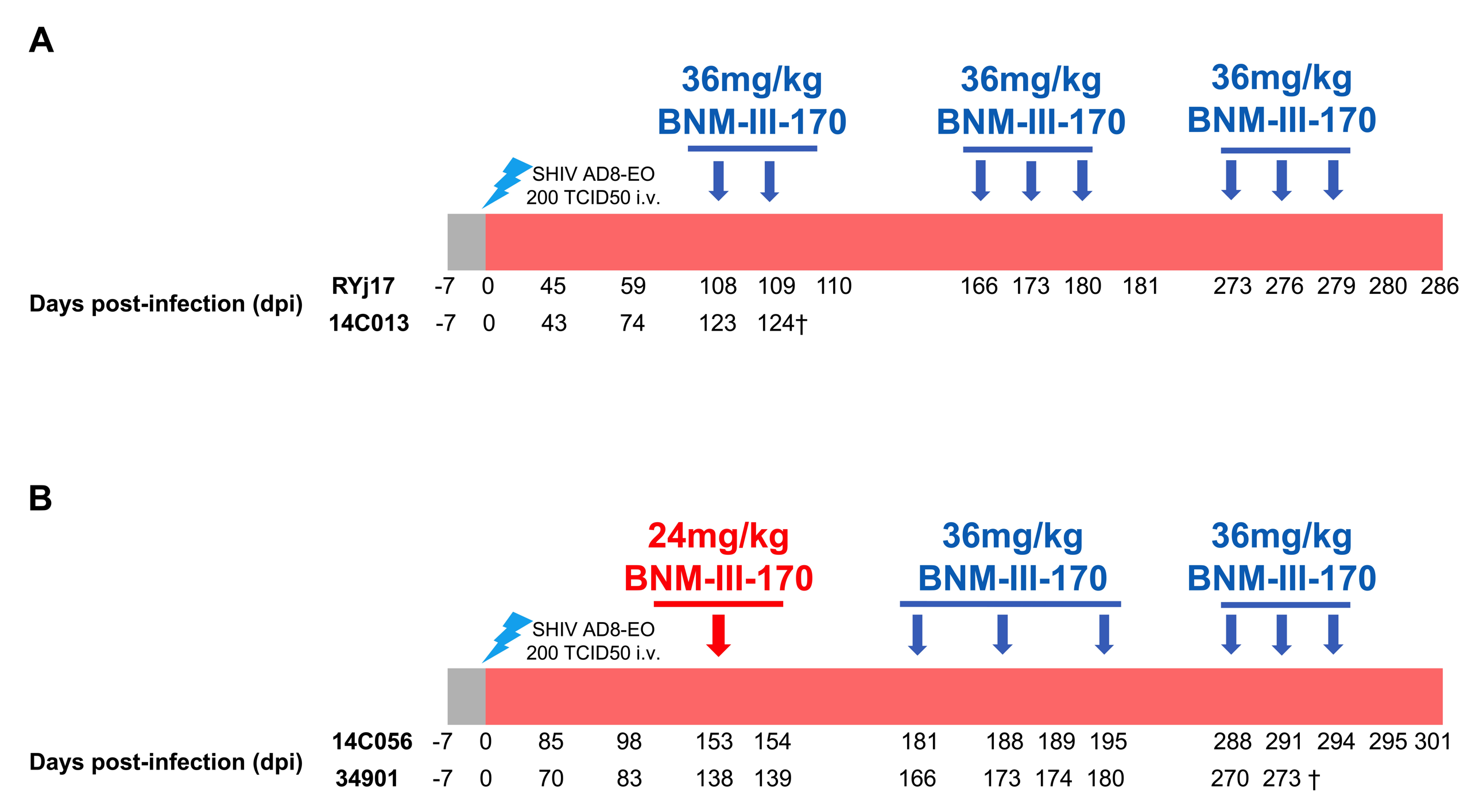

Supplement: Fig. S3 — Study design by collection group of subcutaneous BNM-III-170 treatment in SHIV AD8-EO-infected RMs. [file jvi.00062-25-s0003.jpg]

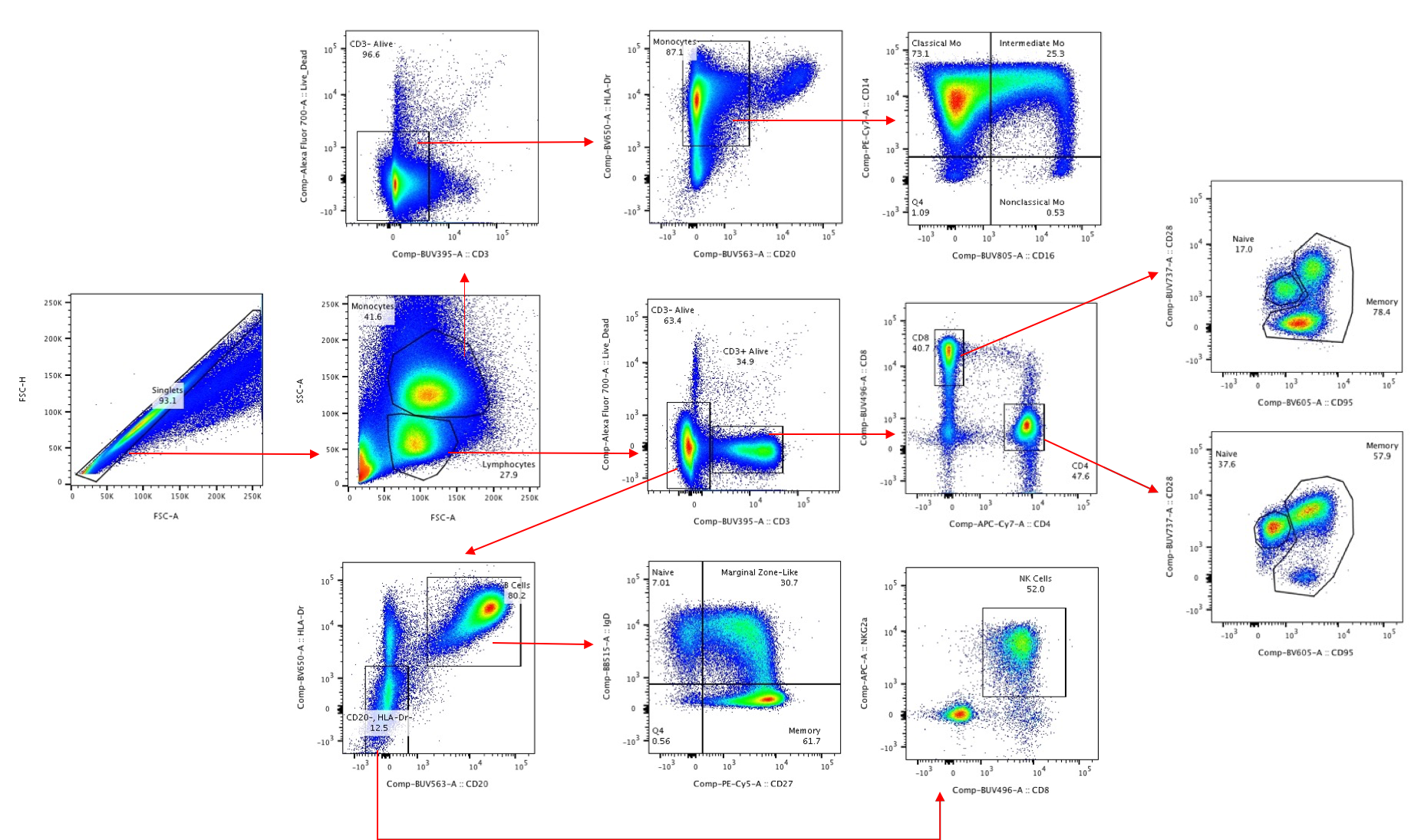

Supplement: Fig. S6 — Flow cytometry gating strategy. [file jvi.00062-25-s0006.tiff]

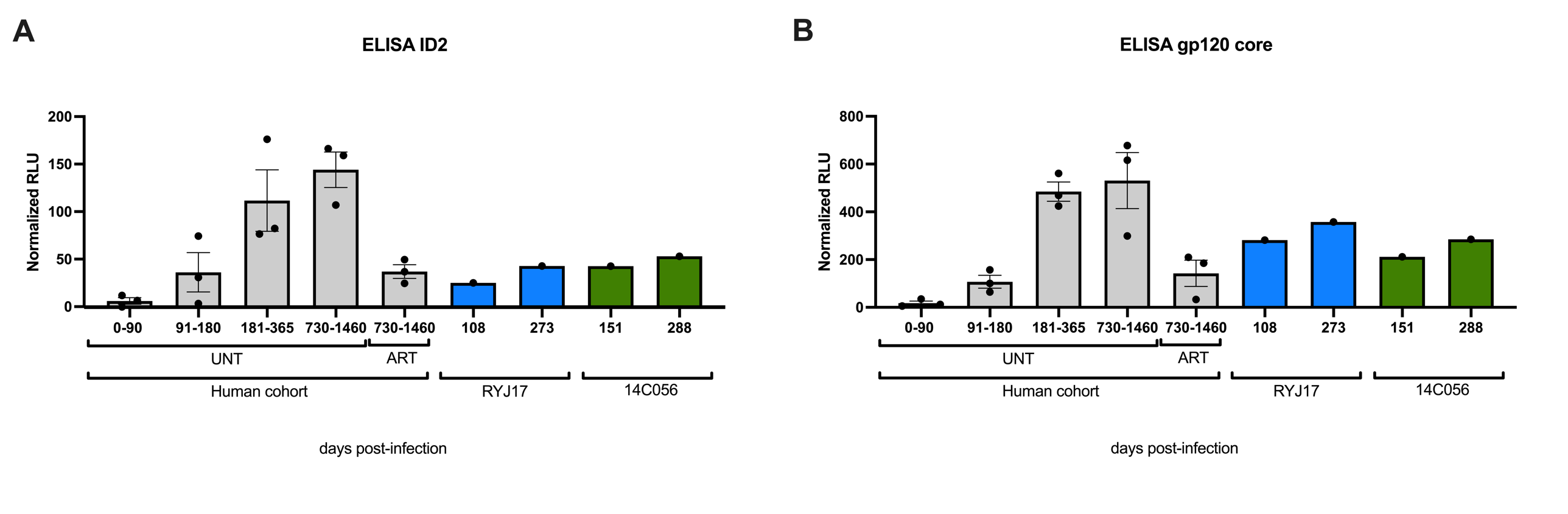

Supplement: Fig. S7 — Comparison of antibody titers in the plasma of PLWH and SHIV AD8-EO-infected RMs. [file jvi.00062-25-s0007.tiff]
