# Supplementary material for: Safety, pharmacokinetics, and biological activity of CD4-mimetic BNM-III-170 in SHIV-infected rhesus macaques
Source: J Virol. 2025 Apr 7;99(5):e00062-25. doi: 10.1128/jvi.00062-25 (PMC12090809; doi:10.1128/jvi.00062-25)
Supplement: Supplemental legends — Legends for Fig. S1 to S7 and Table S1. [file jvi.00062-25-s0008.docx]

**Supplementary Figures and Tables:**

**Figure S1. Enantioselective Synthesis of (+)-(*R,R*,)-BNM-III-170.**

**Figure S2. 17b binding increases with increased concentrations of bioavailable BNM-III-170 in treated, uninfected RMs.** Association between levels of BNM-III-170 in plasma from uninfected, BNM-III-170-treated RMs and 17b binding to CH058TF-infected CEM.NKr-CCR5-sLTR-Luc cells following incubation with plasma from BNM-III-170-treated RMs for **(A)** all tested SQ doses of BNM-III-170 as well as **(B)** 3mg/kg SQ, **(C)** 6mg/kg SQ, **(D)** 12mg/kg SQ, **(E)** 14mg/kg SQ, **(F)** 24mg/kg SQ doses of BNM-III-170 (Spearman correlation).

**Figure S3. Study design by collection group of subcutaneous BNM-III-170 treatment in SHIV AD8-EO-infected RMs.** (A) Group 1 included animals RYj17 and 14C013 and (B) group 2 included animals 14C056 and 34901.

**Figure S4. Longitudinal SHIV plasma viral loads for each animal.** Plasma viral loads (SHIV RNA copies/mL) measured by RT-qPCR longitudinally for all SHIV-infected RMs. The dashed horizontal line represents the assay’s limit of detection (LOD; 60 copies/mL) with undetectable events plotted as 30 copies/mL. The red and blue vertical lines represent 36mg/kg and 24mg/kg doses of BNM-III-170, respectively.

**Fig. S5. Longitudinal frequencies and absolute numbers of lymphocytes following the first dose of each treatment cycle of BNM-III-170.** Frequencies and absolute numbers of lymphocytes were determined by Complete Blood Counts (CBCs). Doses of 36mg/kg are depicted in blue while doses of 24mg/kg are depicted in red.


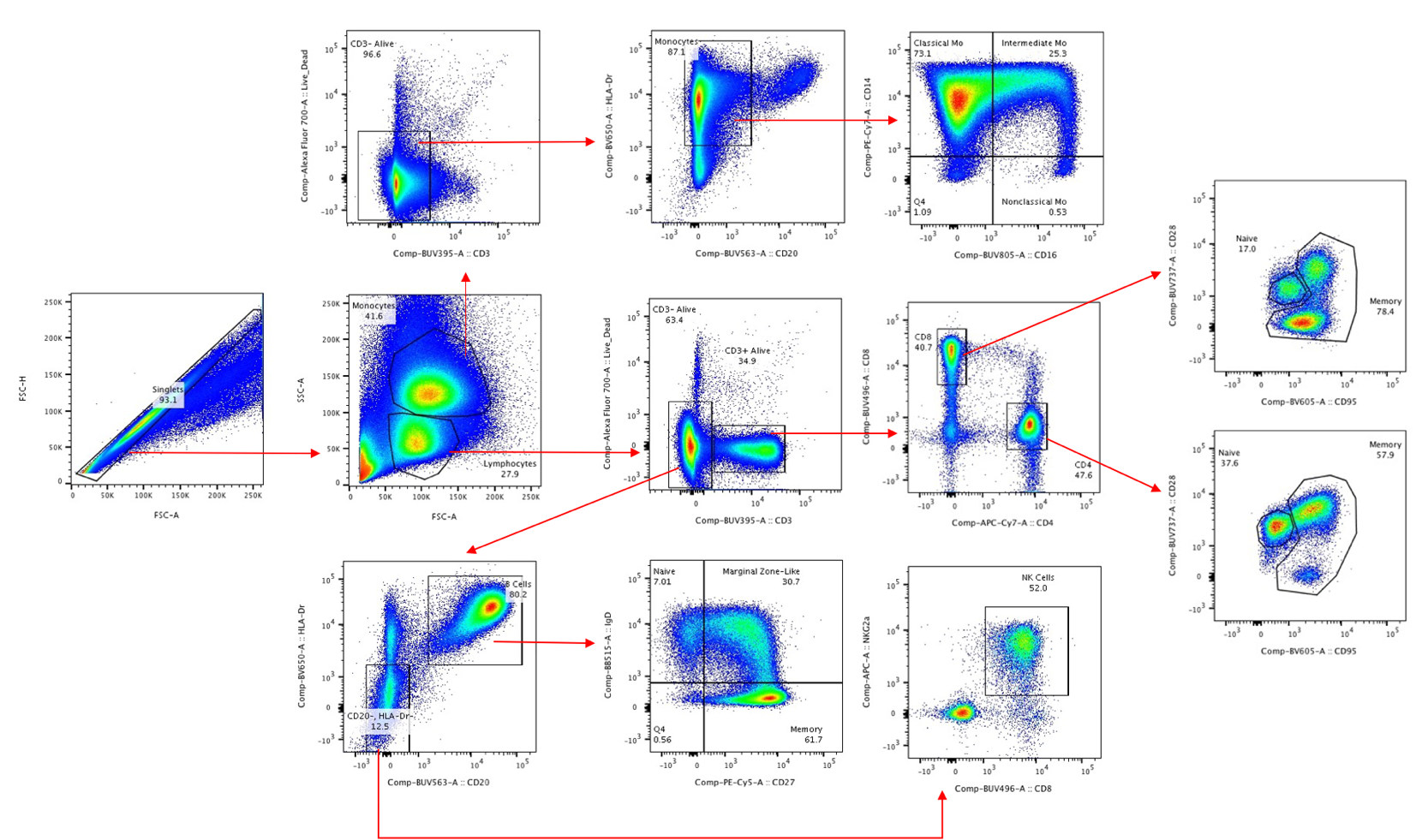


**Figure S6. Flow cytometry gating strategy.** Representative staining with the gating strategy used in PBMCs for T cells, B cells, monocytes, and NK cells (shown in Figure 4B).

**Figure S7. Comparison of antibody titers in the plasma of PLWH and SHIV AD8-EO-infected RMs. (A)** Anti-cluster A antibody titers and **(B)** CD4i antibody titers of PLWH and SHIV AD8-EO-infected RMs at various post-infection timepoints measured via ID2 ELISA and CD4 bound gp120 core ELISA respectively.

**Table S1. Uninfected and SHIV AD8-EO-infected rhesus macaque characteristics.** Animal ID. Infection status. Sex. Age in months at beginning of study. Annotated symbol in figures.
